# Optimizing bandwidth utilization and traffic control in ISP networks for enhanced smart agriculture

**DOI:** 10.1371/journal.pone.0300650

**Published:** 2024-03-25

**Authors:** Emad S. Hassan, Ayman E. A. Abdelaal, Ahmed S. Oshaba, Atef El-Emary, Moawad I. Dessouky, Fathi E. Abd El-Samie

**Affiliations:** 1 Department of Electrical Engineering, College of Engineering, Jazan University, Jizan, Saudi Arabia; 2 Department of Electronics and Electrical Communication, Faculty of Electronic Engineering, Menoufia University, Menouf, Egypt; 3 Department of Information Technology, College of Computer and Information Sciences, Princess Nourah bint Abdulrahman University, Riyadh, Saudi Arabia; University of the West of Scotland, UNITED KINGDOM

## Abstract

As the demand for high-bandwidth Internet connections continues to surge, industries are exploring innovative ways to harness this connectivity, and smart agriculture stands at the forefront of this evolution. In this paper, we delve into the challenges faced by Internet Service Providers (ISPs) in efficiently managing bandwidth and traffic within their networks. We propose a synergy between two pivotal technologies, Multi-Protocol Label Switching—Traffic Engineering (MPLS-TE) and Diffserv Quality of Service (Diffserv-QoS), which have implications beyond traditional networks and resonate strongly with the realm of smart agriculture. The increasing adoption of technology in agriculture relies heavily on real-time data, remote monitoring, and automated processes. This dynamic nature requires robust and reliable high-bandwidth connections to facilitate data flow between sensors, devices, and central management systems. By optimizing bandwidth utilization through MPLS-TE and implementing traffic control mechanisms with Diffserv-QoS, ISPs can create a resilient network foundation for smart agriculture applications. The integration of MPLS-TE and Diffserv-QoS has resulted in significant enhancements in throughput and a considerable reduction in Jitter. Employment of the IPv4 header has demonstrated impressive outcomes, achieving a throughput of 5.83 Mbps and reducing Jitter to 3 msec.

## 1. Introduction

Smart agriculture, often referred to as precision agriculture, represents a transformative paradigm in modern farming practices. It leverages cutting-edge technologies to enhance crop yield, resource efficiency, and overall sustainability. In smart agriculture, a diverse array of tools including Internet-of-Things (IoT), sensors, drones, satellite imagery, and data analytics are employed to collect and analyze real-time data from fields [1–5]. This data-driven approach enables farmers to make informed decisions, optimizing irrigation schedules, fertilization, pest control, and harvesting times. By tailoring agricultural practices to the specific needs of different sections of a field, smart agriculture minimizes resource wastage and environmental impact, while maximizing productivity. This paradigm shifts towards data-driven decision-making and not only boosts efficiency but also holds the potential to address the global challenges of food security in the face of growing population and changing climate conditions. The success of smart agriculture critically hinges on robust and high-bandwidth connectivity to facilitate seamless data transmission between various devices and central management systems [6–10].

The rapid escalation in the demand for high-bandwidth Internet connections and applications is propelled by the diverse spectrum of Internet usage for both business and entertainment needs. Internet Service Providers (ISPs) are compelled to optimize their internal networks, focusing on bandwidth utilization and resource management. To address the intricate challenges surrounding bandwidth utilization within ISP networks, various technologies have been introduced. Two prominent technologies in this regard are Diffserv Quality of Service (Diffserv-QoS) and Multi-Protocol Label Switching-Traffic Engineering (MPLS-TE) [11, 12]. These technologies are synergistically combined to achieve superior performance, efficient resource management, and effective bandwidth utilization.

ISPs must deploy various technologies to ensure the satisfaction of their customers’ requirements, when delivering services. One of the primary challenges faced by ISPs pertains to bandwidth utilization and consumption within their networks [13]. In cases multiple paths exist within the provider network leading to a specific destination, the routing protocol plays a crucial role in selecting the optimal path. Numerous routing protocols, such as Open Shortest Path First (OSPF), Intermediate System to Intermediate System (IS-IS), Routing Information Protocol (RIP), and Border Gateway Protocol (BGP), are available, each with its own metric for path selection [14, 15]. These metrics vary; some, like RIP, depend on hop count, while others, like IS-IS, involve a configurable cost value. More complex attributes characterize protocols such as BGP, while those like OSPF base metric calculation on bandwidth. However, even protocols utilizing bandwidth as their metric encounter a significant issue. They often rely on the overall configured or assumed default bandwidth of a particular interface rather than its actual utilized bandwidth. This can result in the protocol selecting a path with a higher configured bandwidth (e.g., 100 Mbps) as the best option, directing all traffic through it. Meanwhile, another link with a slightly lower bandwidth (e.g., 90 Mbps) remains underutilized, because it is not considered the best path. This discrepancy can lead to congestion on the favored link and inefficient use of the underutilized link.

MPLS-TE addresses the previously mentioned issue by overriding the default behavior of routing protocols [16]. It selects the optimal path for a specific destination, often referred to as Forwarding Equivalent Class (FEC), based on the available (un-reserved) bandwidth on network links and the required bandwidth for that FEC. While this model can influence the selection of the best path using reserved and un-reserved bandwidth, it lacks the ability to limit or control the actual user traffic. This limitation may result in congestion if, for instance, the FEC is defined as 10 Mbps, but users generate a traffic load of 50 Mbps, leading to data queuing or dropping [17].

Although MPLS-TE is effective in reserving expected bandwidth for each FEC, it does not exert control over users’ real traffic that might surpass the designated limits. In response to this challenge, Diffserv-QoS plays a crucial role. Diffserv-QoS offers a solution to avoid or manage congestion, essentially controlling the real traffic of users through a multi-step process involving classification, marking, policing, shaping, and scheduling.

In the context of smart agriculture, bandwidth management and traffic control play a pivotal role in ensuring timely and accurate data transmission for tasks such as precision irrigation, livestock monitoring, and crop health assessment. The symbiotic relationship between MPLS-TE and Diffserv-QoS can empower ISPs to not only provide high-quality services to the agriculture sector but also support the seamless integration of data-intensive applications that drive the efficiency, productivity, and sustainability of modern farming practices [6].

This paper delves into the surging demand for high-bandwidth Internet connectivity, propelled by diverse business and entertainment needs. ISPs face the challenge of optimizing internal network bandwidth usage and resource management. The synergy between two pivotal technologies, Diffserv-QoS and MPLS-TE, emerges as a solution to enhance performance, resource allocation, and bandwidth efficiency within ISPs. The paper addresses the intricate bandwidth consumption challenges faced by ISPs, scrutinizing the role of routing protocols in selecting optimal paths among multiple network options. Despite protocols using bandwidth as a metric, they often rely on configured interface bandwidth, leading to congestion and underutilization. MPLS-TE provides an alternative by considering un-reserved bandwidth on network links and FEC requirements, optimizing path selection. However, it lacks control over user traffic, risking congestion. In this interplay, Diffserv-QoS becomes crucial, managing congestion through classification, marking, policing, shaping, and scheduling. The paper explores how MPLS-TE and Diffserv-QoS collaboratively address bandwidth challenges, offering ISPs comprehensive tools to optimize allocation, while regulating actual traffic for an efficient user experience. In summary, beyond addressing the technical aspects of bandwidth optimization and traffic control in ISP networks, this paper underscores the direct relevance of these concepts in shaping the landscape of smart agriculture. By establishing a strong link between network technologies and agricultural advancements, the paper paves the way for a more connected, efficient, and technologically empowered agricultural sector.

## 2. Quality of Service (QoS) model

Converged networks necessitate QoS management for the diverse array of applications sharing a common network infrastructure [18]. It becomes essential to discern and treat traffic from distinct applications, uniquely. This special treatment ensures optimal performance for individual applications, even amidst congestion or queuing delays. Effective control of user bandwidth usage is imperative. For instance, applications like voice and video, which are sensitive to factors like Jitter and packet loss, require equitable access despite competition from regular data traffic.

QoS plays a pivotal role by categorizing packets into various traffic classes, enabling the allocation of appropriate resources based on distinct criteria. An extensively adopted mechanism for this purpose is Diffserv, which facilitates traffic management and QoS provision in contemporary IP networks [19, 20]. In this landscape, addressing the issue of "unfairness" is paramount.

### 2.1 Quality of Service (QoS) metrics

A range of metrics gauge the QoS in a network, with the most significant ones outlined below [21, 22]:

1. Bandwidth: It reflects the end-to-end capacity to carry information.
2. Delay: It represents the time taken for information delivery across the network.
3. Delay variation (Jitter): It represents for fluctuations in end-to-end delays due to packet queuing.
4. Loss: It quantifies the percentage of undelivered packets, commonly linked to congestion.

Enhancement of these parameters can be approached through various means. While expanding link bandwidth is a possibility, it often entails considerable expenses. Another strategy involves data and header compression, albeit application-specific. In the present landscape, advanced queuing strategies within the interface software queue have gained prominence. Some prevalent techniques include [21]:

- MDRR (Modified Deficit Round Robin)
- WFQ (Weighted Fair Queuing)
- CBWFQ (Class-Based Weighted Fair Queuing)
- LLQ (Low-Latency Queuing)

Two distinct definitions have emerged to encapsulate QoS techniques: Integrated Services and Differentiated Services.

### 2.2 Integrated services

Integrated services represent the initial foray by the Internet Engineering Task Force (IETF) into expanding IP functionalities beyond basic best-effort services [23]. This endeavor employed RSVP signaling to communicate precise QoS requisites to the network. The central concept revolves around routers reserving resources across the network, initiating a connection reminiscent of circuit-switched call setup. However, the practical implementation of this model was hindered by scalability concerns, preventing its widespread deployment.

### 2.3 Differentiated Services (DiffServ)

The fundamental concept behind DiffServ is the establishment of Per Hop Behavior (PHB) predicated on prior data markings, all without necessitating circuit establishment. This model has emerged as the most widely-adopted approach for achieving QoS enhancements [24–26].

### 2.4 QoS framework

**Fig 1:**
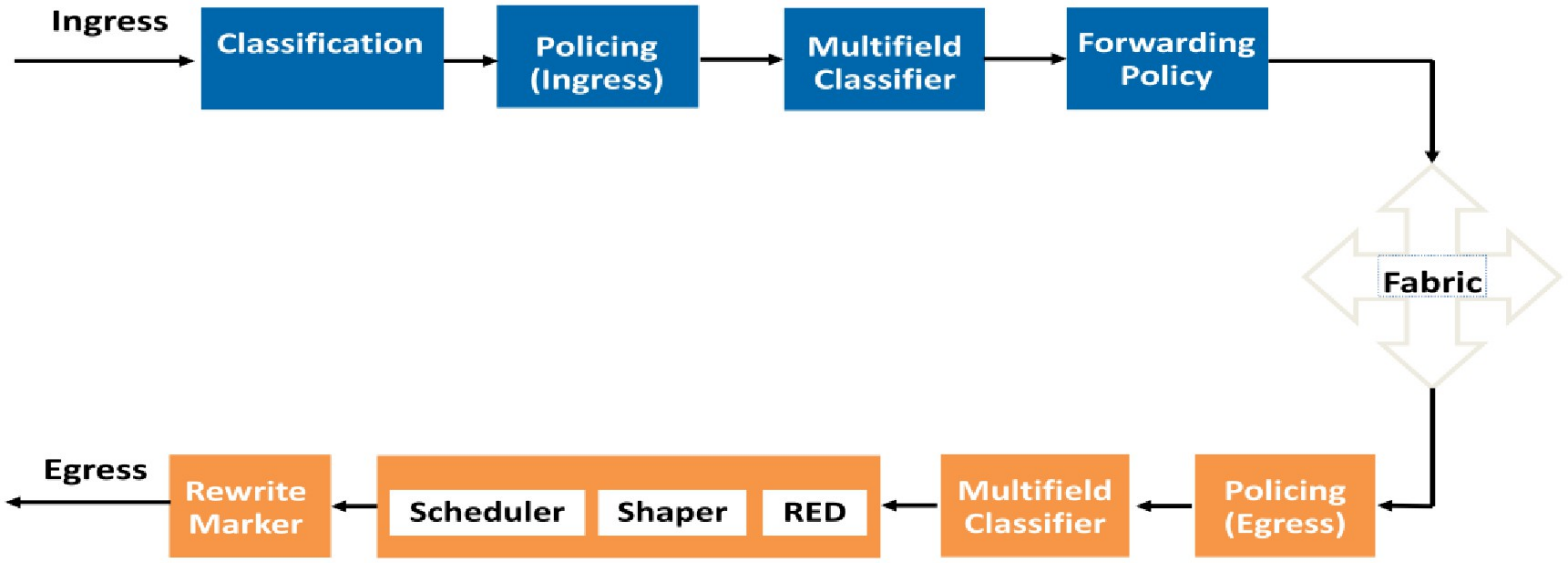
QoS model.

The QoS model, as depicted in Fig 1, is broadly adopted for data QoS determination. The initial step entails classifying incoming traffic into multiple categories based on applications such as Data, Voice, Video, and Network Control. Classification is achieved through filters applied at the interfaces, considering various parameters like source or destination IP addresses, port numbers, and protocol identifiers. Following classification, each class is marked with a value denoting its significance. This marking transpires at the Access Layer, situated closest to users, while the Core Layer devices subsequently respond accordingly based on these markings. Further insights into the marking process are discussed in the subsequent section [24].

### 2.5 Marking

**Fig 2:**
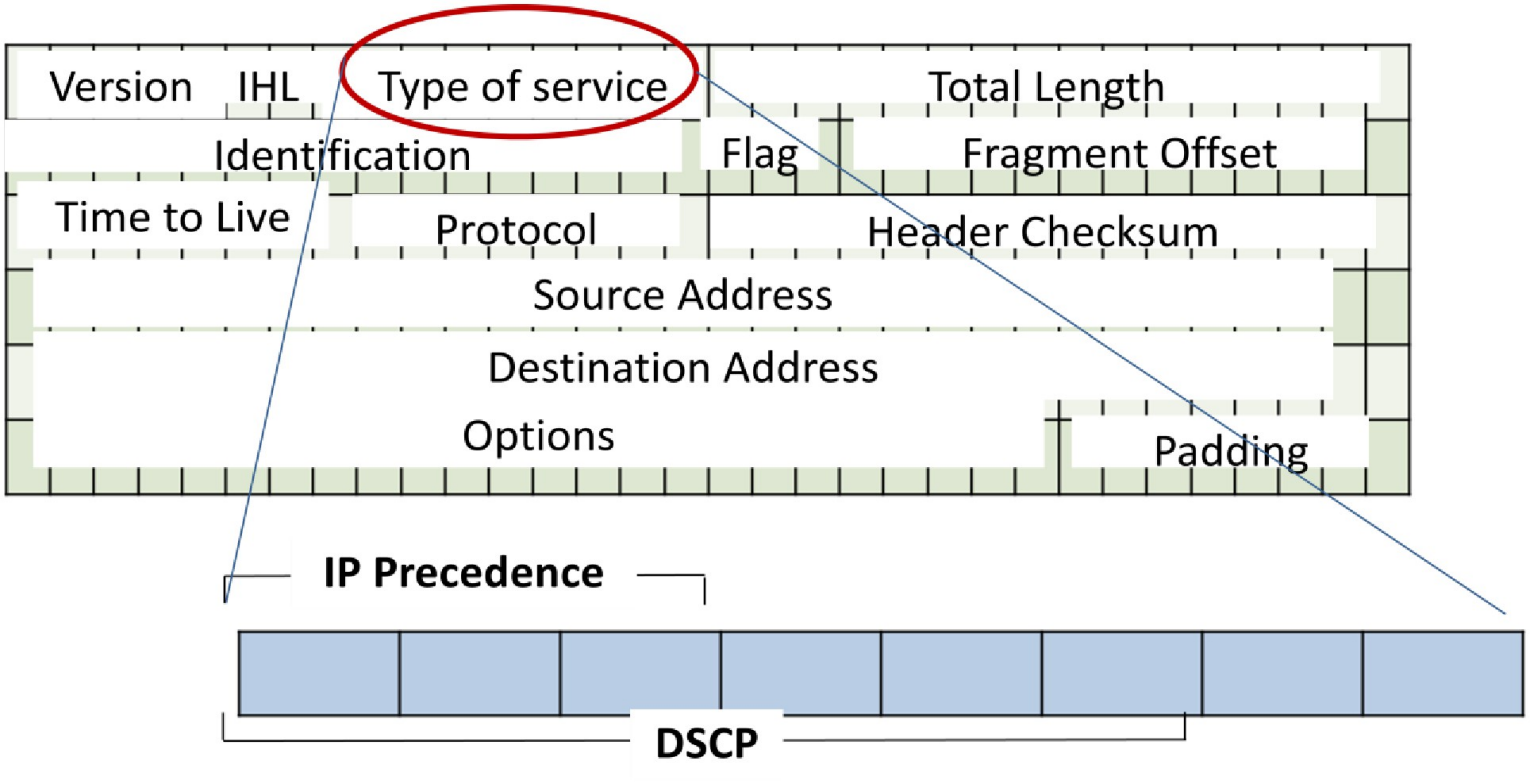
IPv4 header and ToS field.

**Fig 3:**
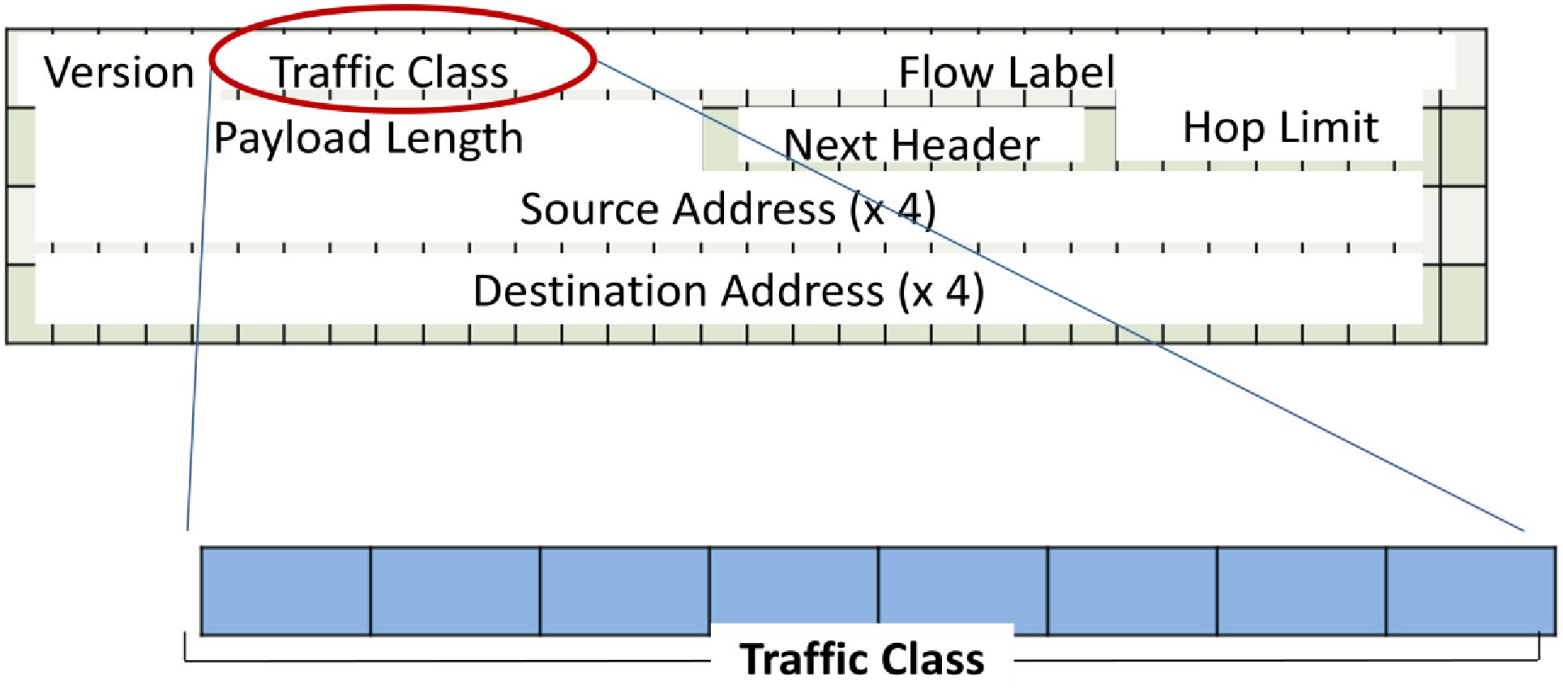
IPv6 header and ToS location.

**Fig 4:**
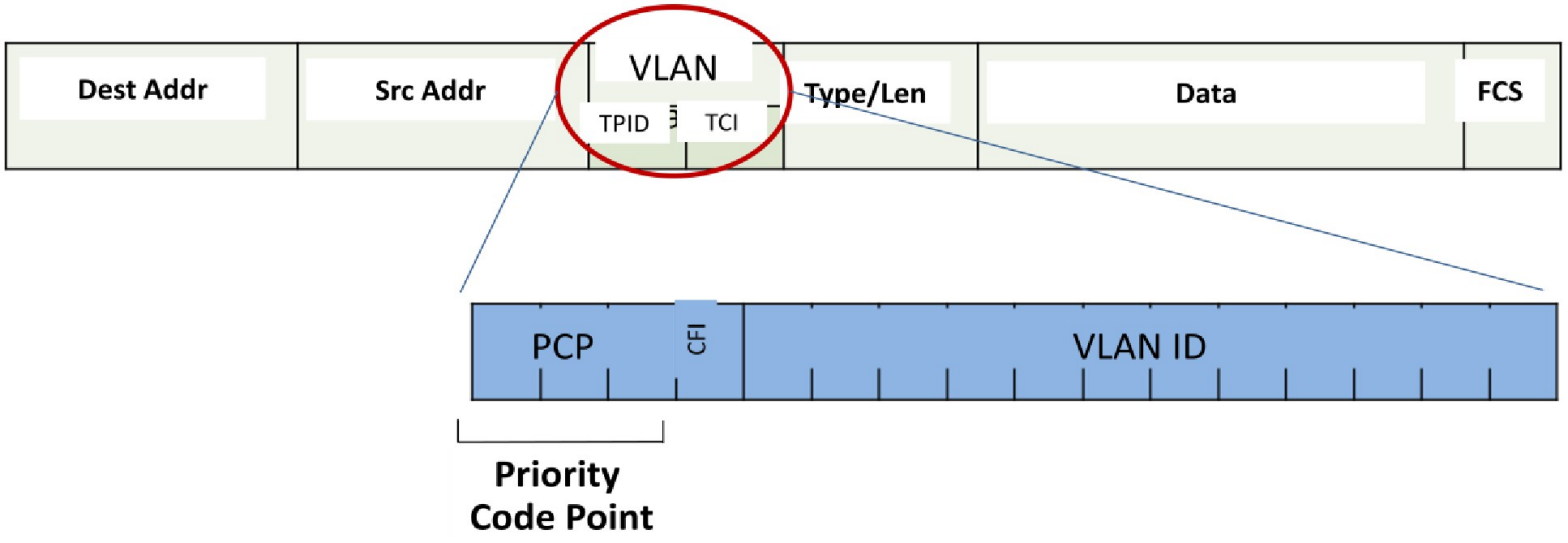
IEEE 802.1Q and CoS field location.

**Fig 5:**
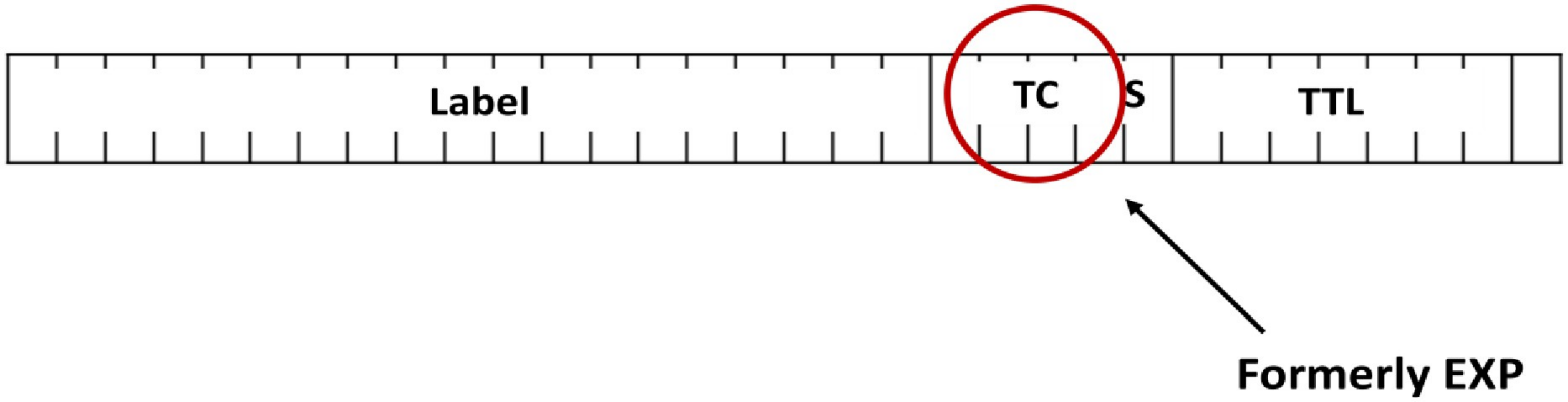
MPLS header and the location of EXP bits.

Marking is a crucial step that occurs at one or multiple layers within the data stack, including the IPv4 header, IPv6 header, 802.1Q Tag, or MPLS header. The process is illustrated in Figs 2 to 5. The IPv4 header, detailed in Fig 2, features the Type of Service (ToS) field, responsible for QoS. ToS is represented by a 6-bit value, encoded in either IP precedence or DiffServ Code Point (DSCP) formats, as shown in Fig 3 for the IPv6 header. In Fig 4, the IEEE 802.1Q header introduces the Class of Service (CoS), presenting 8 QoS values across 3 bits. This tag is integral for VLAN segmentation within Ethernet frames, often referred to as "Trunking" [27–29]. The MPLS header, illustrated in Fig 5, incorporates the "Experimental Bits" responsible for carrying QoS information.

### 2.6 Policing

**Fig 6:**
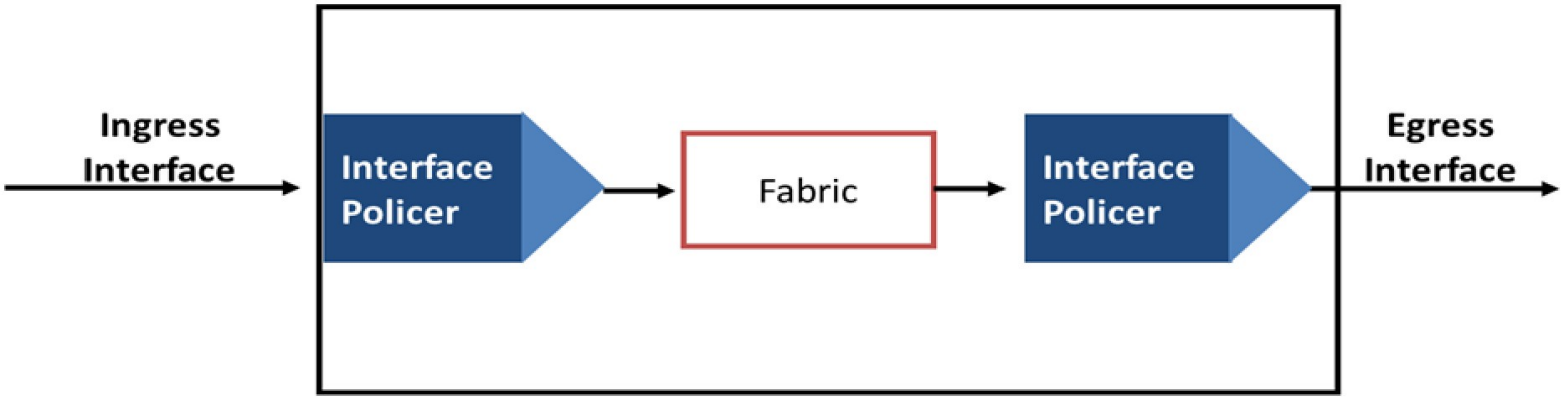
The policing process.

Policing functions to regulate both traffic volume and burstiness, maintaining the QoS-Level of Service Agreement (LSA). It involves marking or discarding excessive traffic and can be implemented at ingress, egress, or both points. Fig 6 offers a comprehensive overview of the policing process [30].

### 2.7 Schedulers

Schedulers determine the prioritization attributes of forwarding classes (queues), constituting a vital facet of QoS implementation [31]. Numerous parameters govern this process:

- Transmission rate (Guaranteed and maximum rates).
- Queue priority.
- Delay buffer (Storage space for traffic bursts).
- Congestion management and avoidance.
  - Support for Random Early Detection (RED) for equitably random traffic dropping.
  - Support for Weighted RED (WRED) for preferential, weighted traffic dropping.

## 3. Proposed hybrid model

Considering the insights gleaned from the preceding sections, it becomes evident that solely employing MPLS-TE within a network offers advantages in efficient resource utilization by reserving requisite bandwidth for each data flow. However, this approach falls short in controlling actual traffic that may surpass anticipated requirements. Conversely, DiffServ-QoS can effectively manage traffic volumes on specific interfaces, ensure defined Service Level Agreements (SLAs), or prioritize distinct traffic types. Nonetheless, it does not inherently enhance the overall resource utilization of network links, thereby potentially contributing to congestion issues. Consequently, the proposed model advocates a combined utilization of both technologies to harness their respective strengths, synergistically.

**Fig 7:**
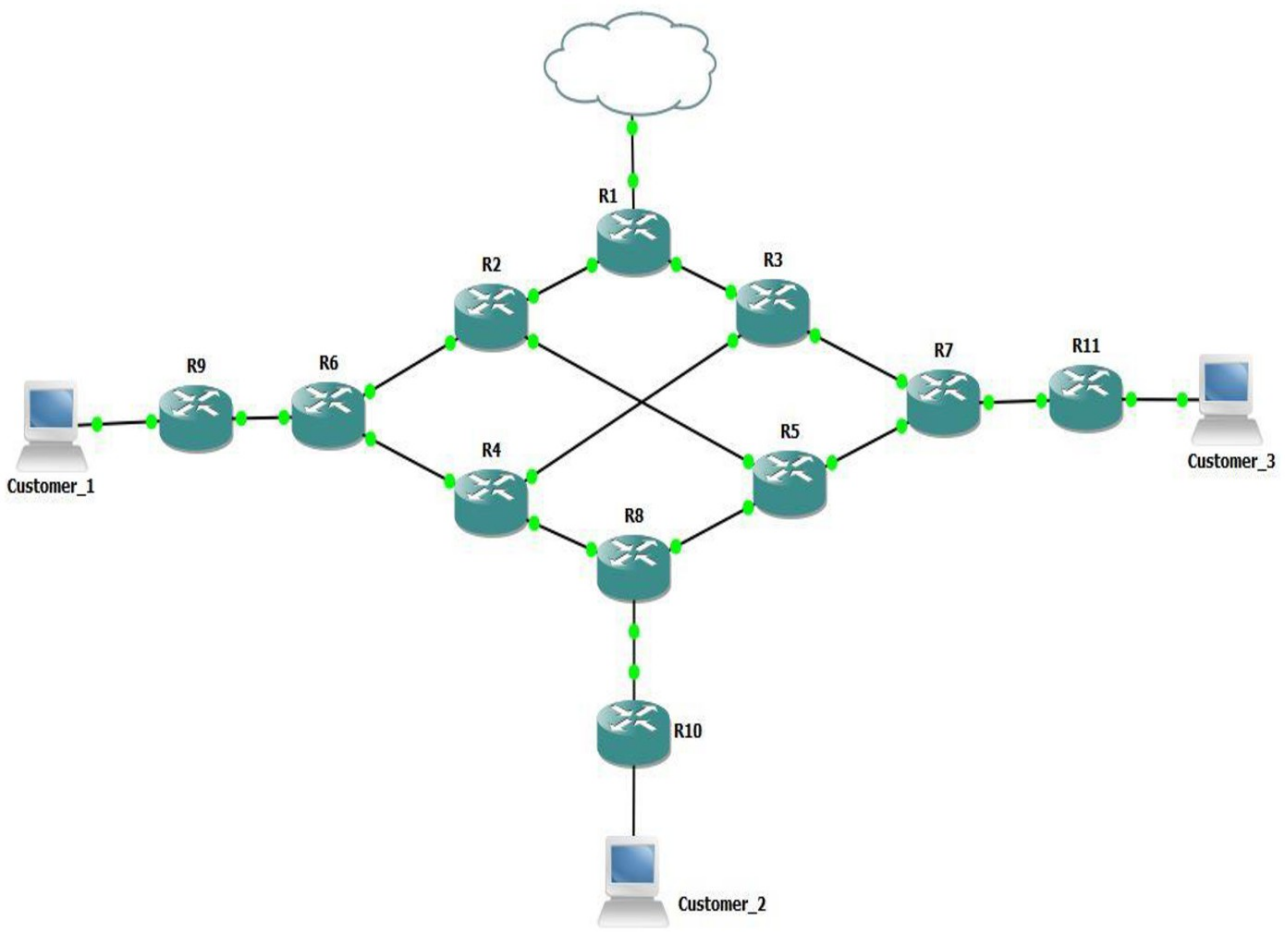
The topology of the case studies.

The ensuing five case studies corroborate the efficacy of this approach in augmenting network performance for simulated voice traffic. The advantages of this proposed model are clearly illustrated in these studies. Illustrated in Fig 7, the accompanying topology underpins the scenarios examined in these case studies.

### 3.1 Topology explanation

The depicted topology represents a streamlined Service Provider (SP) network configuration encompassing 3 Provider Edge (PE) routers, namely R6, R7 and R8 alongside 4 Provider (P) routers (R2, R3, R4, and R5). The framework also integrates 3 Customer Edge (CE) switches: R9, R10 and R11, each linked to data sources and/or voice sources.

The essential components of the network are as follows.

1. Customer Edge Routers (R9, R10, & R11) are Cisco 2600 Models.
2. Provider Edge Routers (R6, R7, & R8) are Cisco 3600 Models.
3. Provider Core Routers (R1, R2, R3, R4, & R5) are Cisco 7200 Models.
4. GNS3 serves as the emulation software, facilitating the operation of actual router Operating Systems (OSs) in a virtualized environment.
5. Cisco Operating System IOS version 12.2 is utilized.
6. Virtualized Windows XP instances are deployed on customer-side devices, employing Hping for TCP background traffic generation and UDP voice traffic.
7. The Jperf application is employed for gauging voice traffic performance metrics, encompassing throughput and Jitter analysis.

All these activities occur within a virtualized environment hosted on a system running Windows 10, equipped with the following software and hardware specifications.

**i- Software**
  - GNS3 simulator v 0.8.6
  - Oracle VM VirtualBox 4.3.10
  - VMware Workstation 8
  - Windows XP professional service pack 3
  - JPerf
  - SolarWinds Wan Killer
**ii- Hardware**

The hardware utilized for this endeavor comprises a Packard Bell LAPTOP equipped with an Intel^®^ Core i5 CPU and 4 GB of RAM.

GNS3 is employed due to its proficiency as a software emulation tool, enabling the execution of genuine Juniper router Operating Systems (JunOS) on personal computers. Given Cisco distinguished status as a premier vendor in the realm of data networking, replete with potent software and hardware offerings, the selection of Cisco products is rooted in their robust capabilities. VMware is leveraged for customer computers, providing the ability to install virtual OSs atop the host OS. Within this framework, Microsoft Windows XP serves as the guest OS across the three virtual machines. The assessment of traffic attributes is conducted through Jperf, a software utility proficient in bandwidth and Jitter testing. Furthermore, Jperf facilitates the emulation of voice traffic on systems functioning as IP phones. To simulate background traffic originating from customer sites, SolarWinds WAN Killer is harnessed as a source of traffic.

### 3.2 Case studies’ target and scenario

The objective is to achieve optimal bandwidth utilization and minimize Jitter across the network. This entails ensuring efficient data transfer and Jitter control among customer site 1, functioning as the source, and customer sites 2 and 3, the designated destinations. Introducing a background TCP traffic employing 560-byte packet sizes serves to emulate non-prioritized traffic congestion. Additionally, VoIP traffic is simulated using UDP traffic characterized by 137-byte packet sizes, employing the G.711 codec for prioritized traffic representation. Both traffic types will remain active for a duration of 30 seconds, followed by two separate measurements of VoIP throughput and Jitter, conducted with a 10-second interval between them. Table 1 summarizes the considered case studies in this work.

**Table 1:** Case studies’ table.

Number	Case study
1	IPv4 Traditional Routing
2	IPv4 QoS LLQ
3	IPv4 MPLS
4	IPv4 MPLS Diffserv-aware TE with MAM
5	IPv4 MPLS Diffserv-aware TE with RDM
6	IPv4 MPLS Diffserv-aware TE with MAM and QoS LLQ
7	IPv4 MPLS Diffserv-aware TE with RDM and QoS LLQ
8	IPv6 Traditional Routing
9	IPv6 QoS LLQ
10	IPv6 MPLS
11	IPv6 MPLS Diffserv-aware TE with MAM
12	IPv6 MPLS Diffserv-aware TE with RDM
13	IPv6 MPLS Diffserv-aware TE with MAM and QoS LLQ
14	IPv6 MPLS Diffserv-aware TE with RDM and QoS LLQ

The configuration for the five routers employed in the case studies are outlined in the appendices provided in [32].

**1- Case Study 1: IPv4 Traditional Routing:** This case shows the classic IPv4 networking working on routing basis without any enhancements in place.

**Fig 8:**
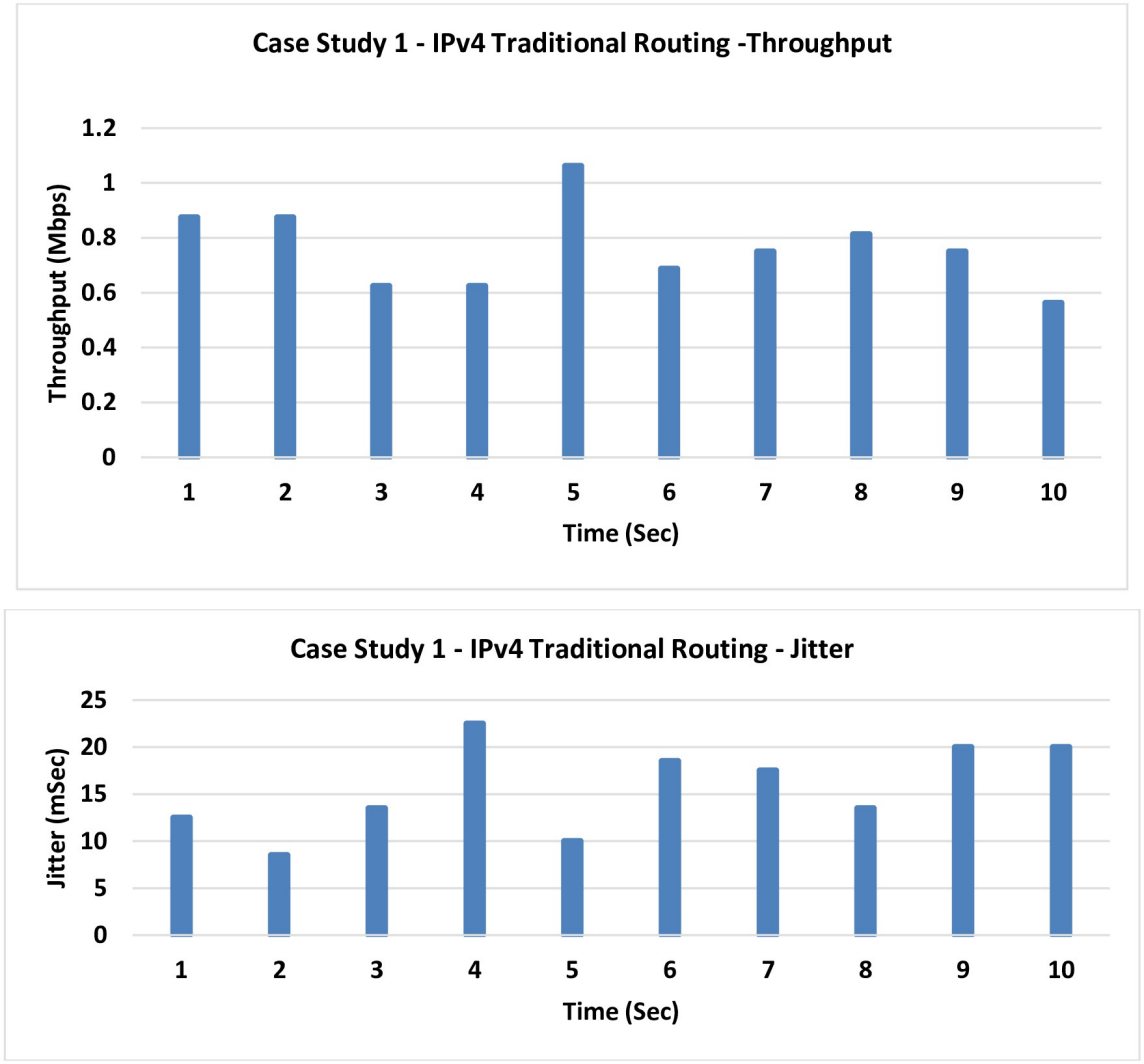
Case Study 1—IPv4 traditional routing.

The results presented in Fig 8 reveal that the average registered throughput is at merely 0.7 Mbps, a notably low figure in comparison to subsequent scenarios. Moreover, the Jitter exhibits a relatively elevated value of 15 milliseconds.

**2. Case Study 2: IPv4 QoS LLQ:** In this case, a level of enhancement is added by running the LLQ.

**Fig 9:**
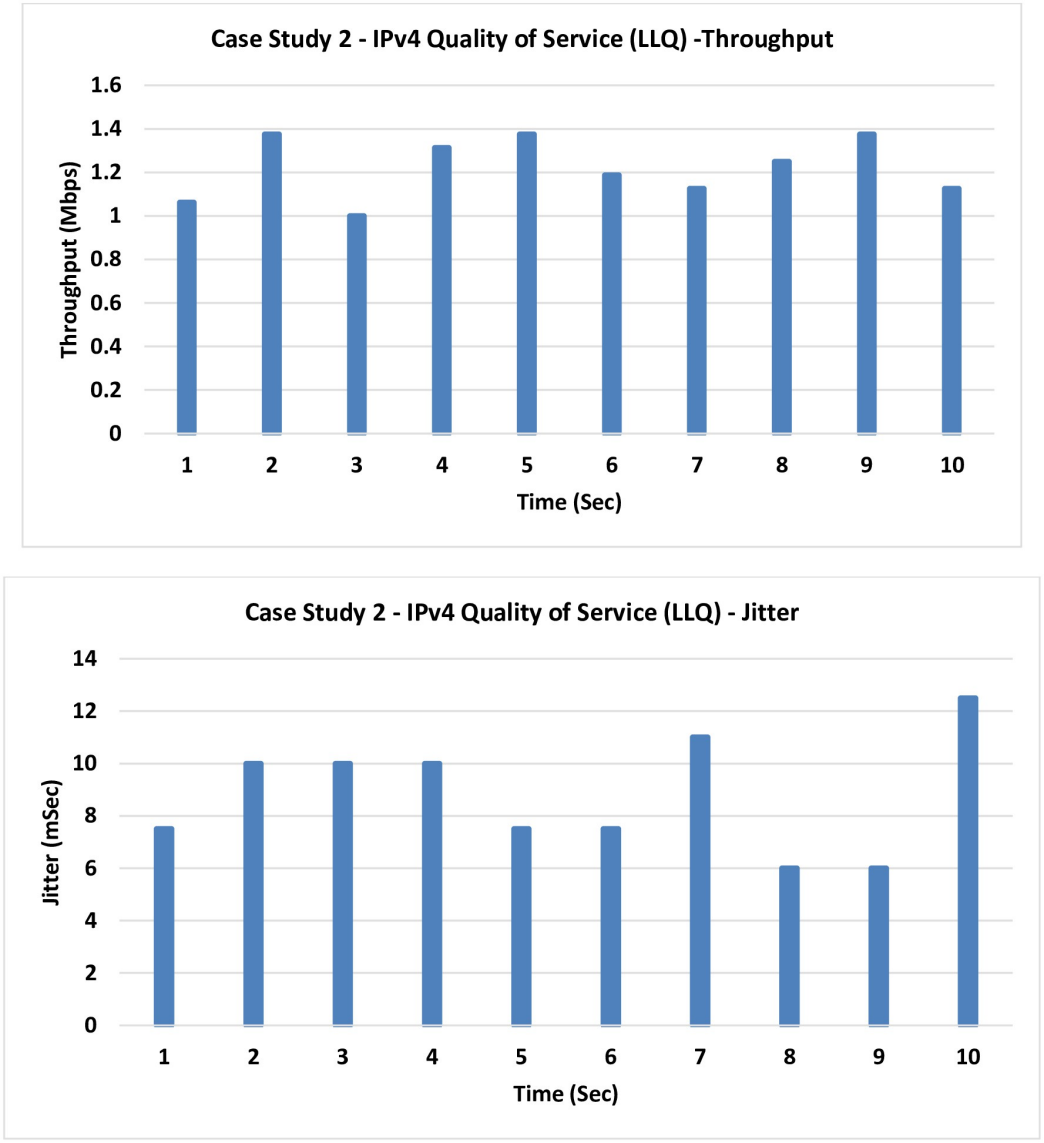
Case Study 2—IPv4 QoS LLQ.

Evident from the results is the minor enhancement introduced by implementing LLQ, impacting both throughput and Jitter as shown in Fig 9.

**3. Case Study 3: IPv4 MPLS:** In this case, the MPLS has been introduced, so forwarding is based on labels on the classical IPv4 routing.

**Fig 10:**
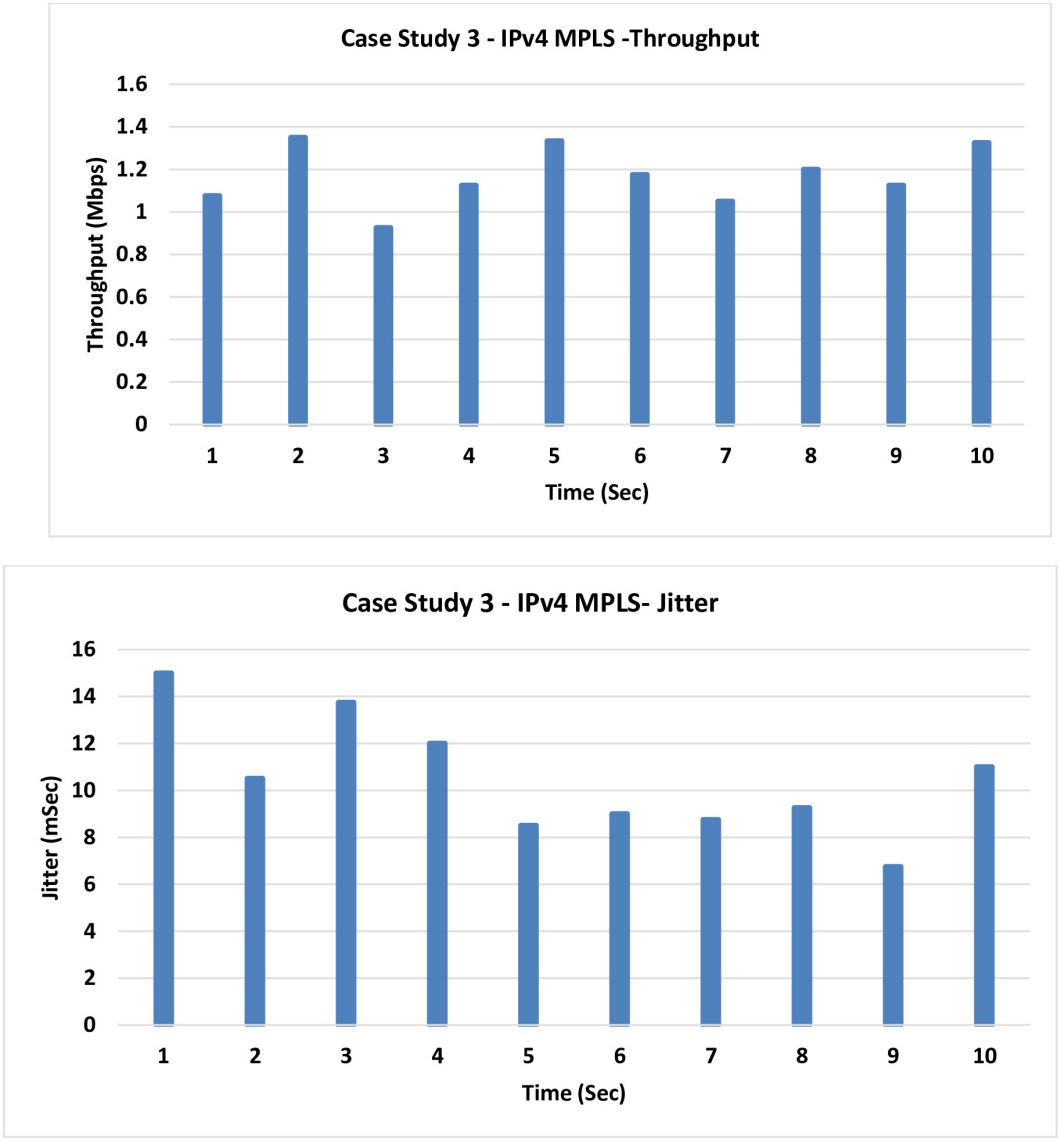
Case Study 3—IPv4 MPLS.

The outcomes in Fig 10 mirror the previous scenario, where a slight improvement is evident upon introducing MPLS. However, in this case, the LLQ component has been omitted.

**4. Case Study 4**
  - **IPv4 MPLS Diffserv-aware TE with MAM:** In this case, the MPLS TE is introduced with the MAM.

**Fig 11:**
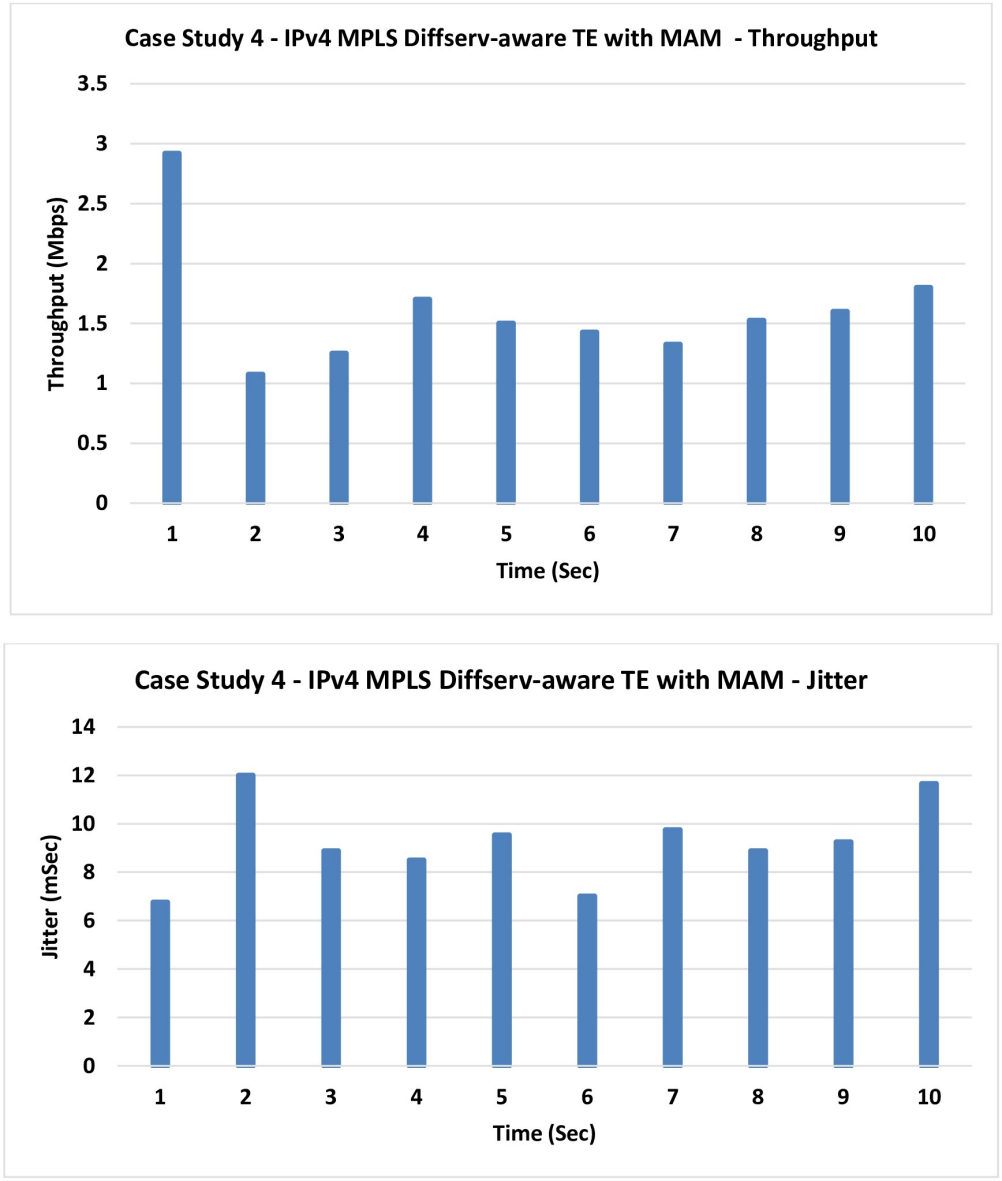
Case Study 4- IPv4 MPLS Diffserv-aware TE with MAM.

No much improvement in the results is noticed as the TE only handles the control plane, but if the data plane is congested–as it is here–no much help can be gained as indicated by Fig 11.

**5. Case Study 5**
  - **IPv4 MPLS Diffserv-aware TE with RDM**: Russian Dolls Model (RDM) is used in this case for the bandwidth reservation instead of the MAM in the previous case.

**Fig 12:**
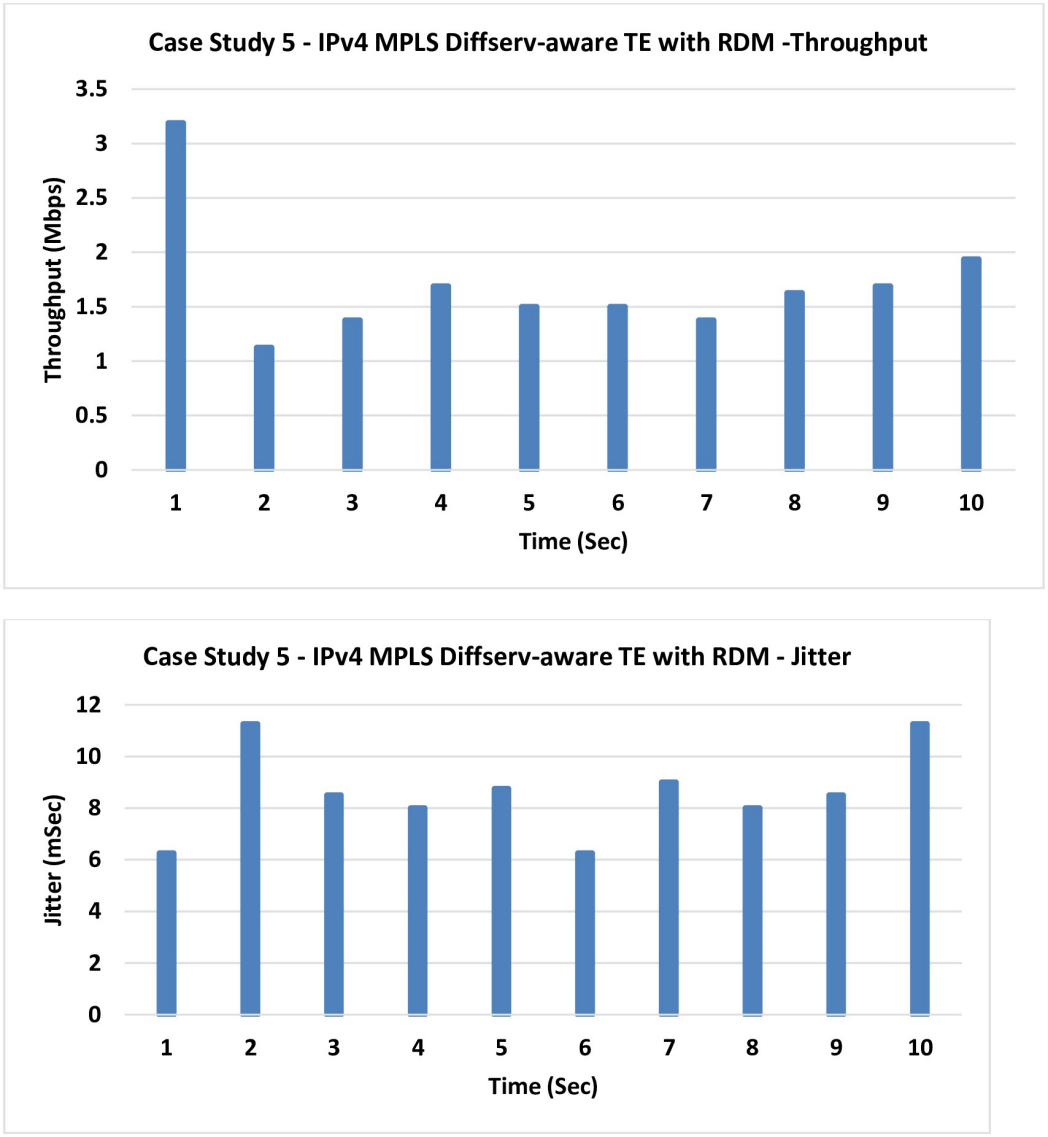
Case Study 5- IPv4 MPLS Diffserv-aware TE with RDM.

The obtained results in Fig 12 are very similar to those of the previous case with a slight improvement as the previously mentioned congestion is still existing, and an avoidance technique is required in the data plane, as the next case introduces.

**6. Case Study 6**
  - **IPv4 MPLS Diffserv-aware TE with MAM and QoS LLQ:** In this case, LLQ as a QoS technique, is tested with MAM as the bandwidth allocation model.

**Fig 13:**
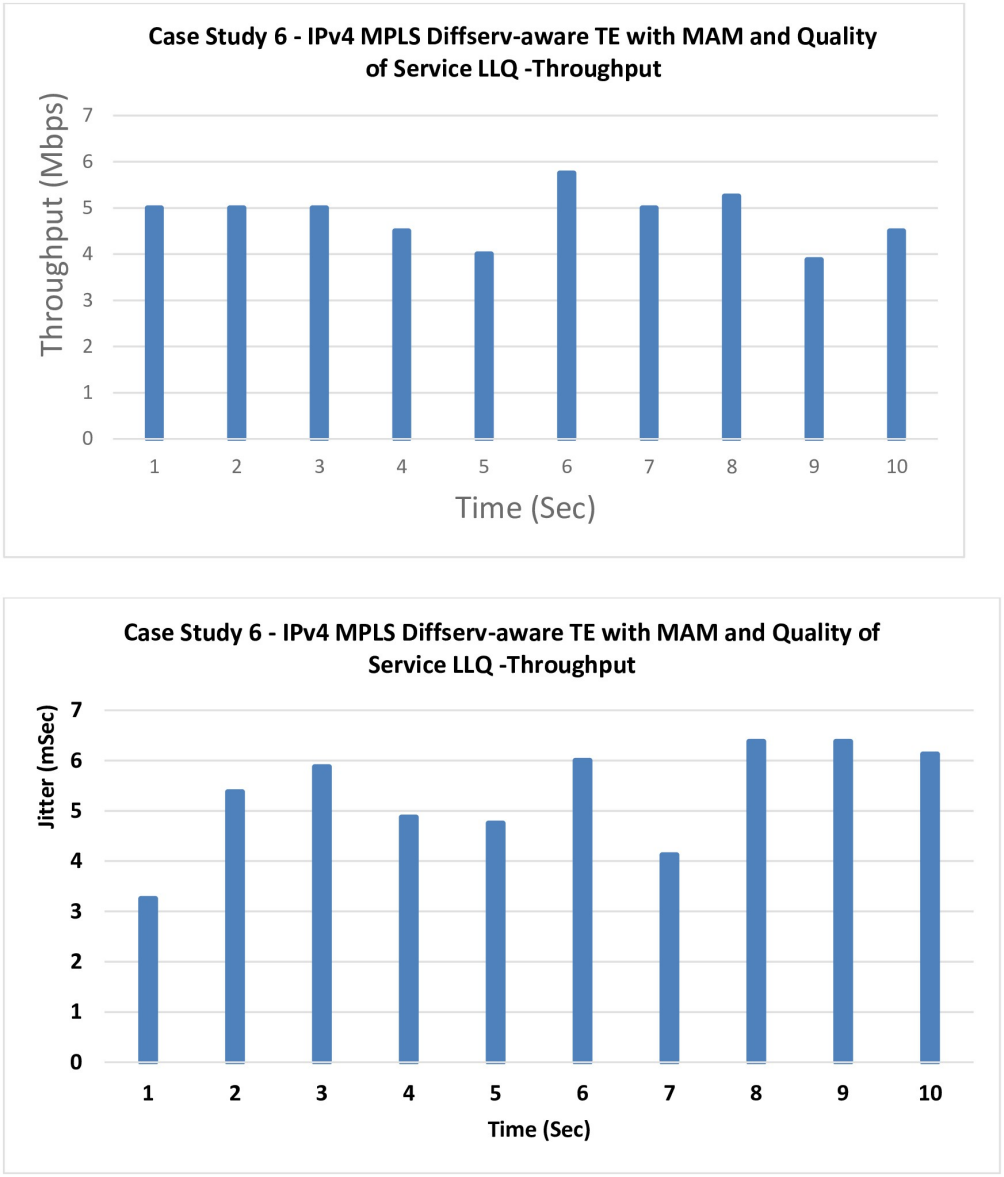
Case Study 6—IPv4 MPLS Diffserv-aware TE with MAM and QoS LLQ.

Fig 13 shows that a significant improvement can be noticed after introducing LLQ, which prioritizes the traffic based on its type (here VoIP is prioritized).

**7. Case Study 7**
  - **IPv4 MPLS Diffserv-aware TE with RDM and QoS LLQ:** In this case, LLQ as a QoS technique, is tested similar to the previous case, but with RDM as the bandwidth allocation model.

**Fig 14:**
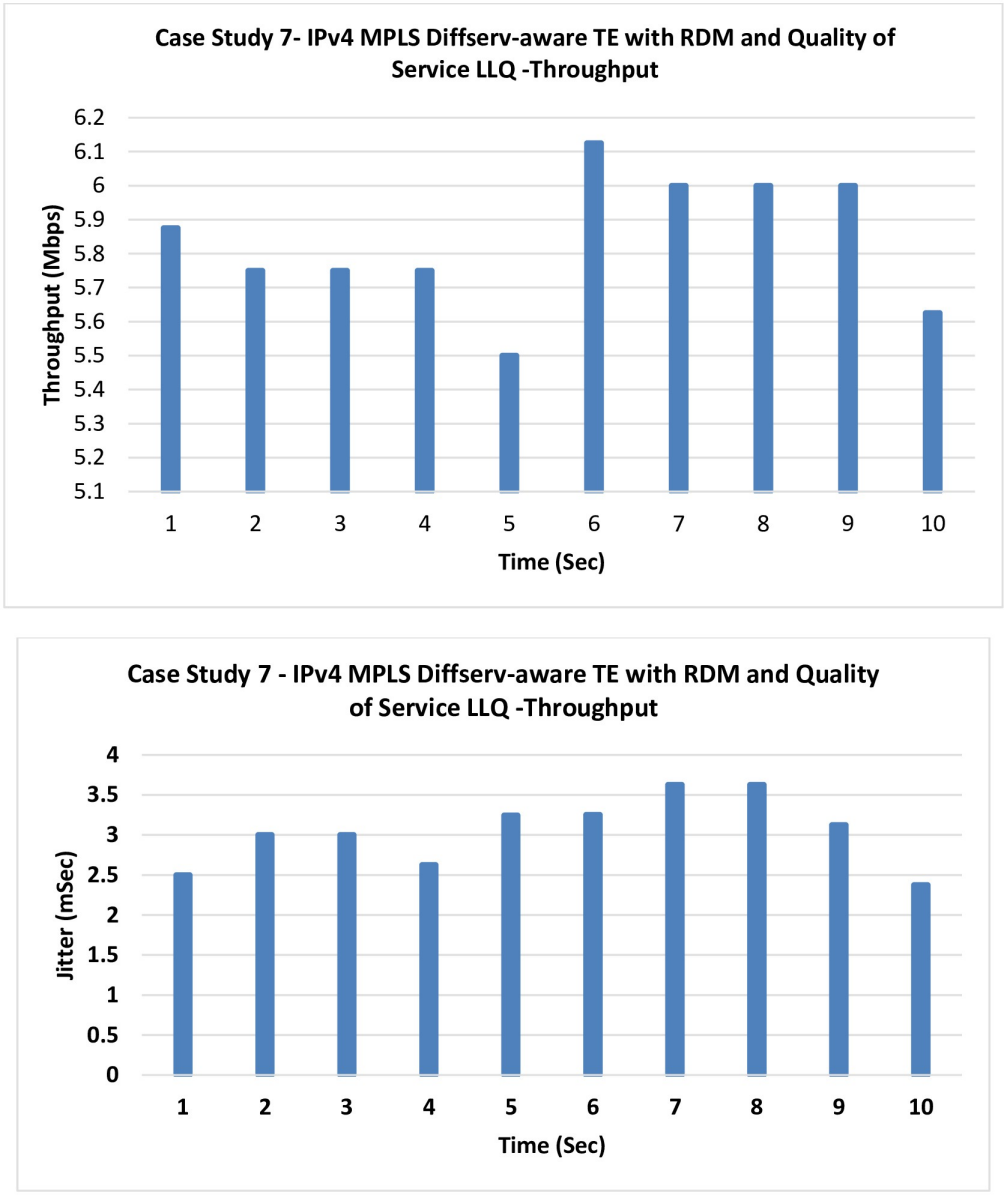
Case Study 7—IPv4 MPLS Diffserv-aware TE with RDM and QoS LLQ.

Fig 14 shows that an additional slight improvement can be noticed in the final case of the IPv4 case studies and this is the proposed model, which is used to merge both MPLS-TE Diffserv-aware using the RDM with the QoS LLQ technique.

**8. Case Study 8**
  - **IPv6 Traditional Routing:** This case shows the classic IPv6 networking working on routing basis without any enhancements in place.

**Fig 15:**
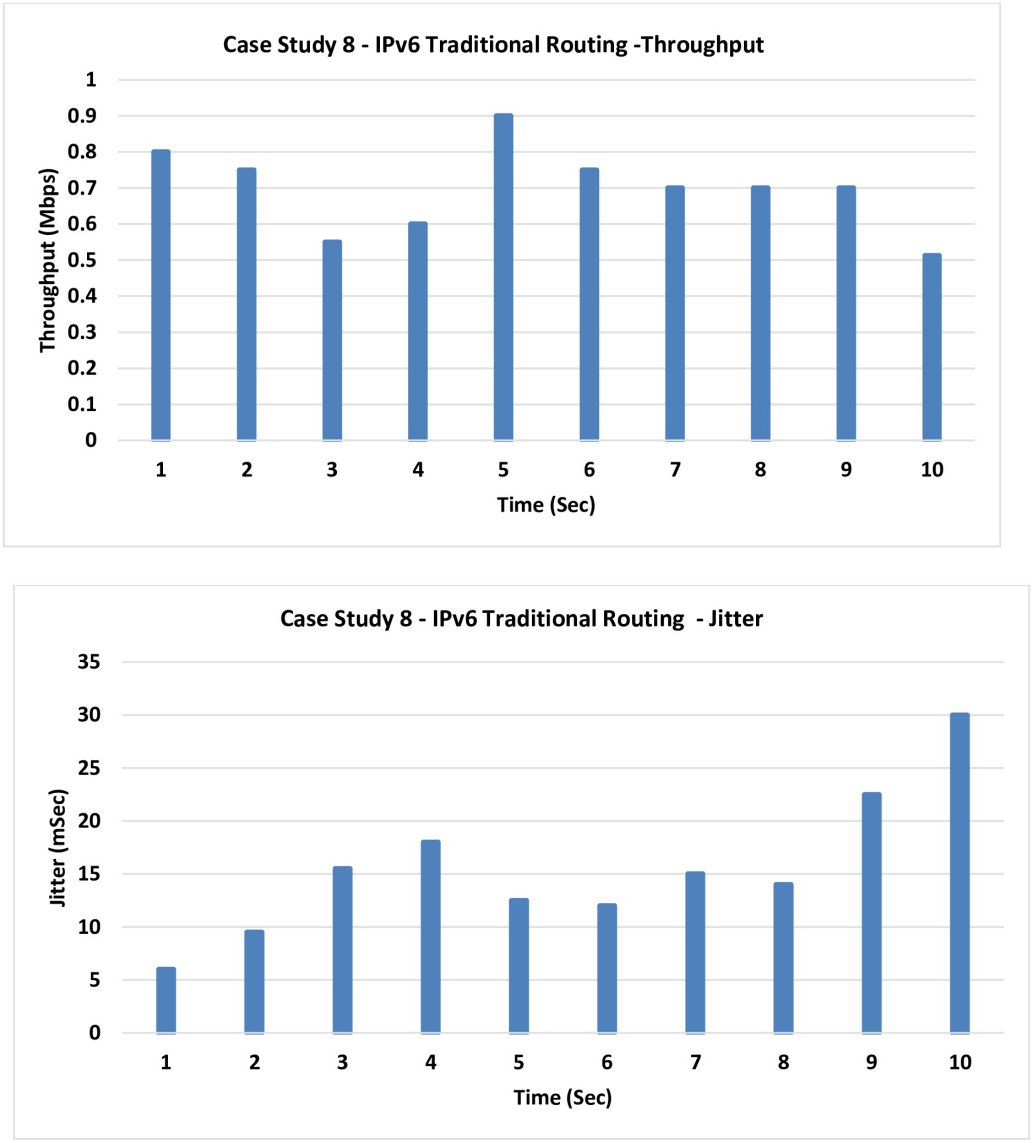
Case Study 8—IPv6 traditional routing.

It is noticed that the average throughput is 0.7 Mbps, which is very low compared to the next cases. Also, the Jitter is relatively high at the value of 15 mSec as indicated in Fig 15.

**9. Case Study 9**
  - **IPv6 QoS LLQ:** In this case, a level of enhancement is added by running the LLQ.

**Fig 16:**
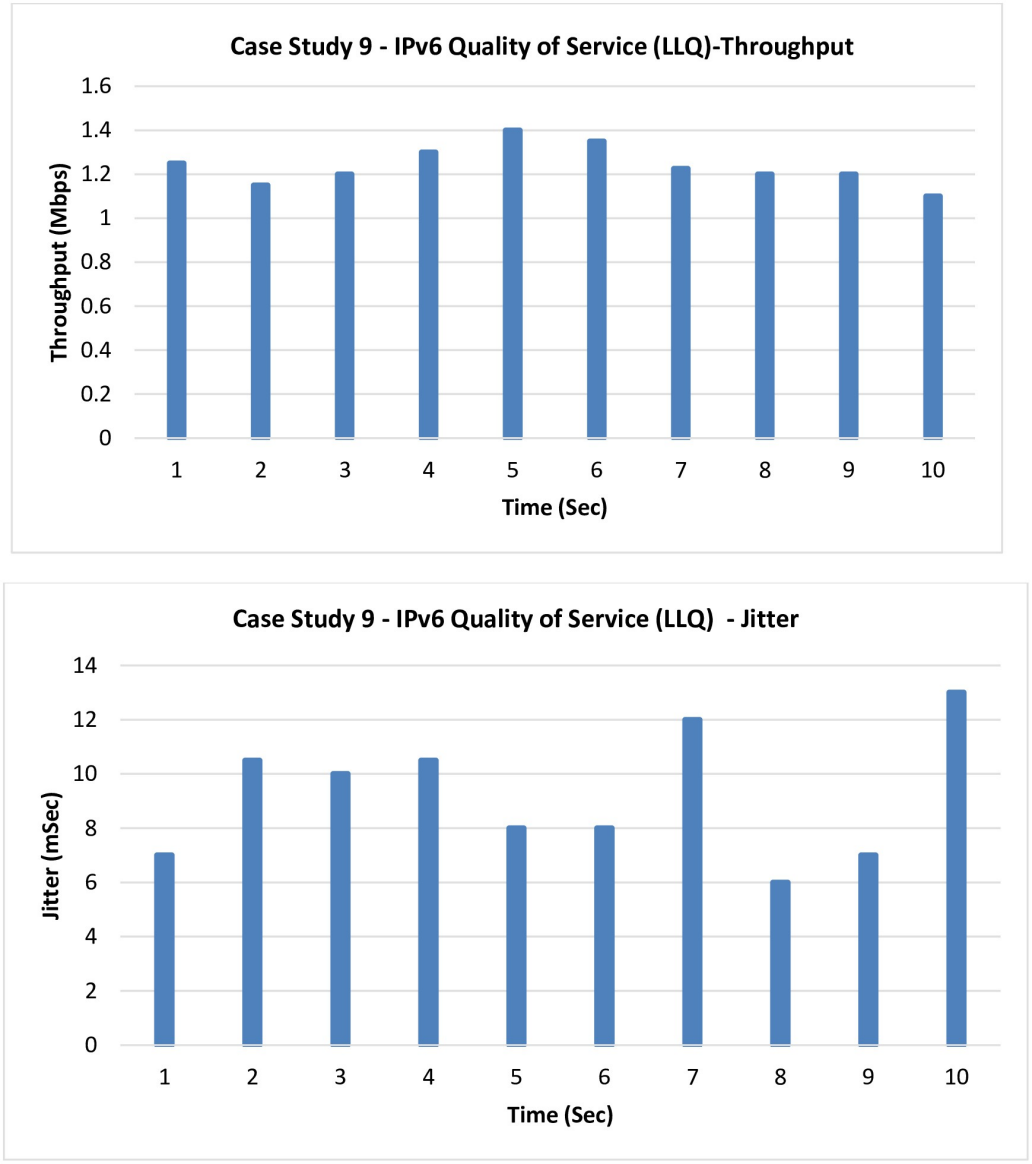
Case Study 9—IPv6 QoS.

From Fig 16, it can be noticed that a little improvement in both the throughput and Jitter has been added by running the LLQ.

**10. Case Study 10**
  - **IPv6 MPLS:** In this case, the MPLS has been introduced, and so forwarding is based on labels on the classical IPv6 routing.

**Fig 17:**
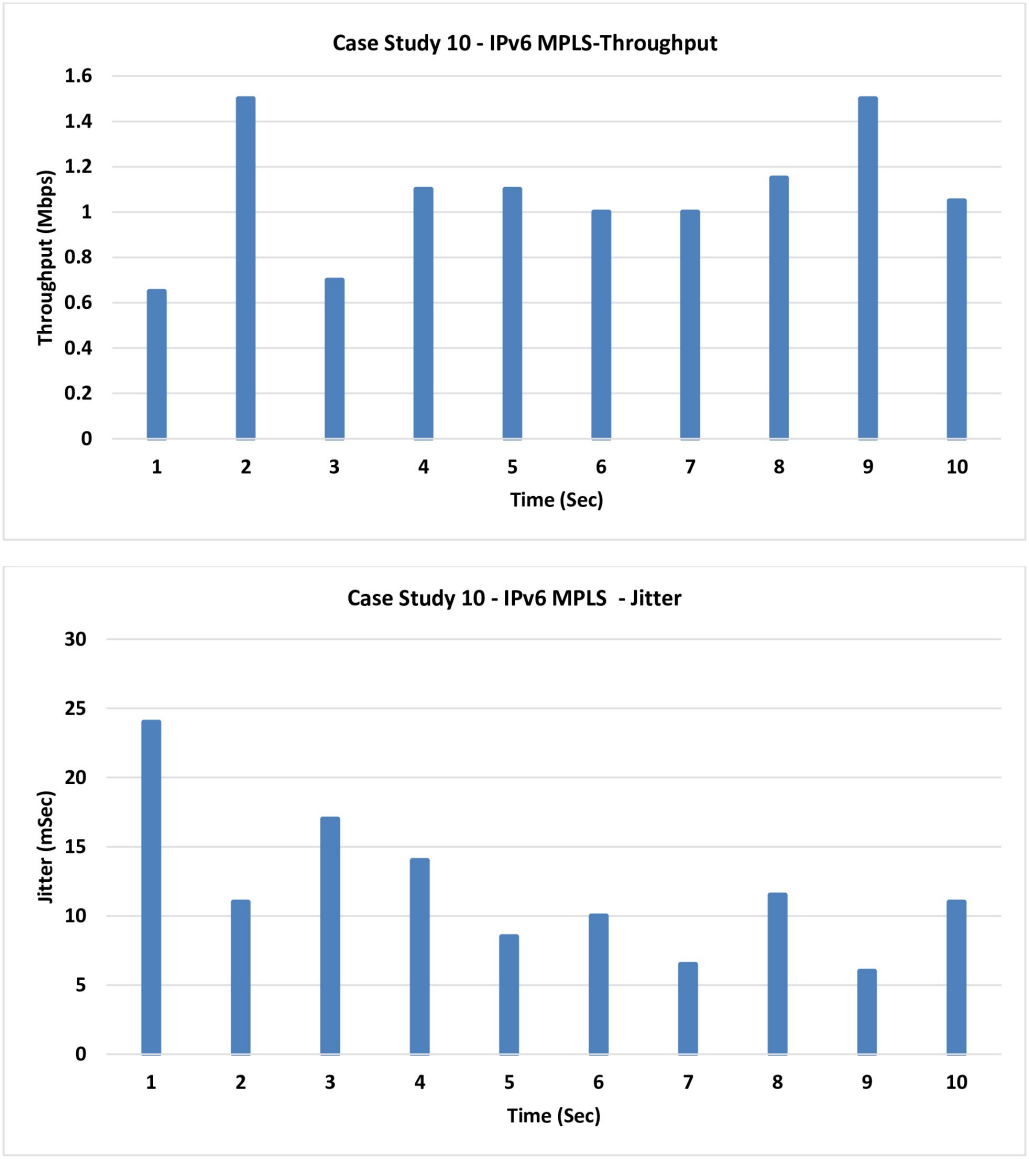
Case Study 10—IPv6 MPLS.

The results presented in Fig 17 are similar to those of the previous case, as here we have added a little improvement by adding the MPLS but the LLQ was removed.

**11. Case Study 11**
  - **IPv6 MPLS Diffserv-aware TE with MAM:** In this case, the MPLS-TE is introduced with the MAM.

**Fig 18:**
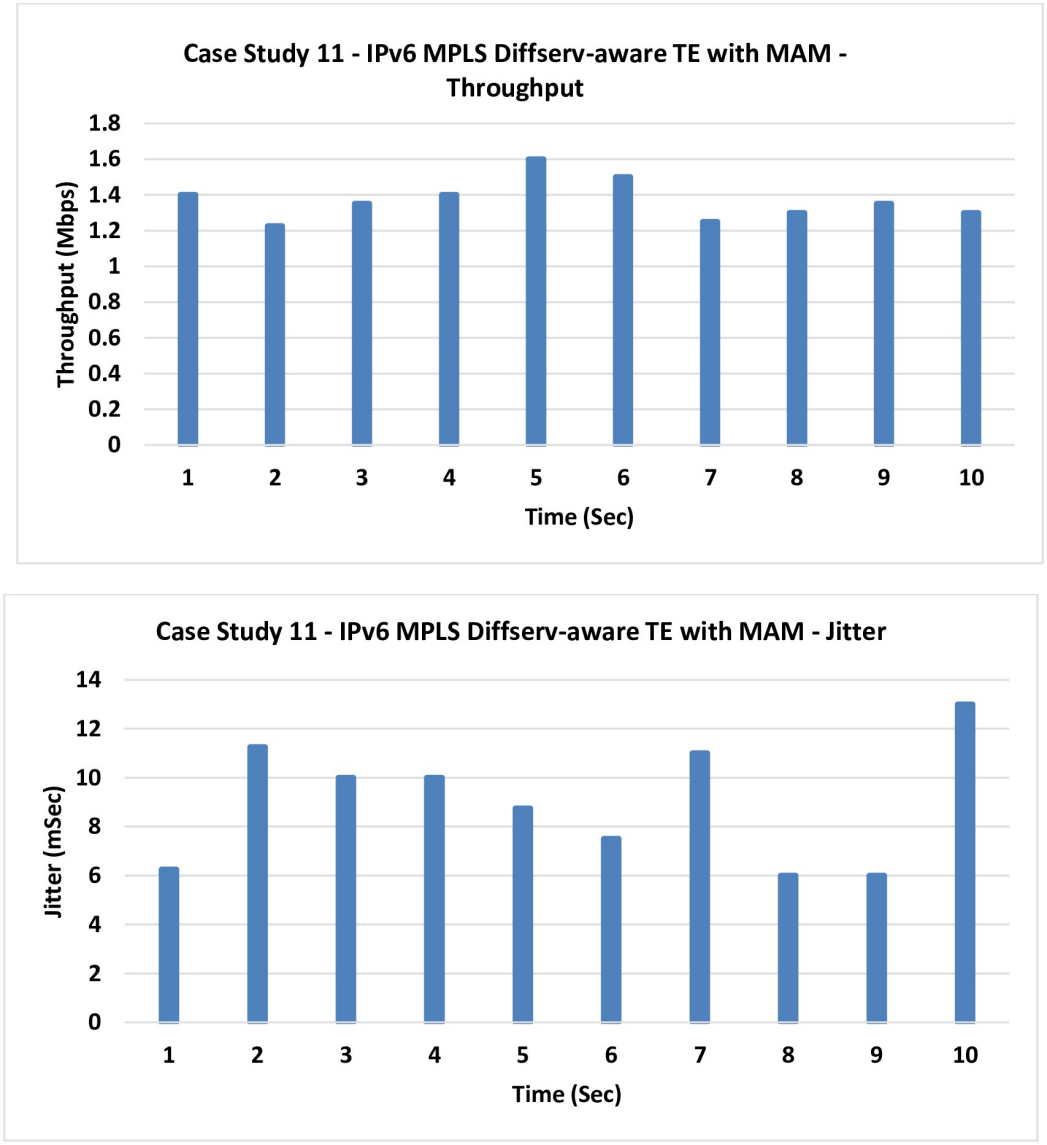
Case Study 11—IPv6 MPLS Diffserv-aware TE with MAM.

No much improvement in results is noticed as the TE only handles the control plane, but if the data plane is congested–as it is here–no much help can be gained as explained in Fig 18.

**12. Case Study 12**
  - **IPv6 MPLS Diffserv-aware TE with RDM:** Russian Dolls Model (RDM) is used in this case for bandwidth reservation instead of the MAM in the previous case.

**Fig 19:**
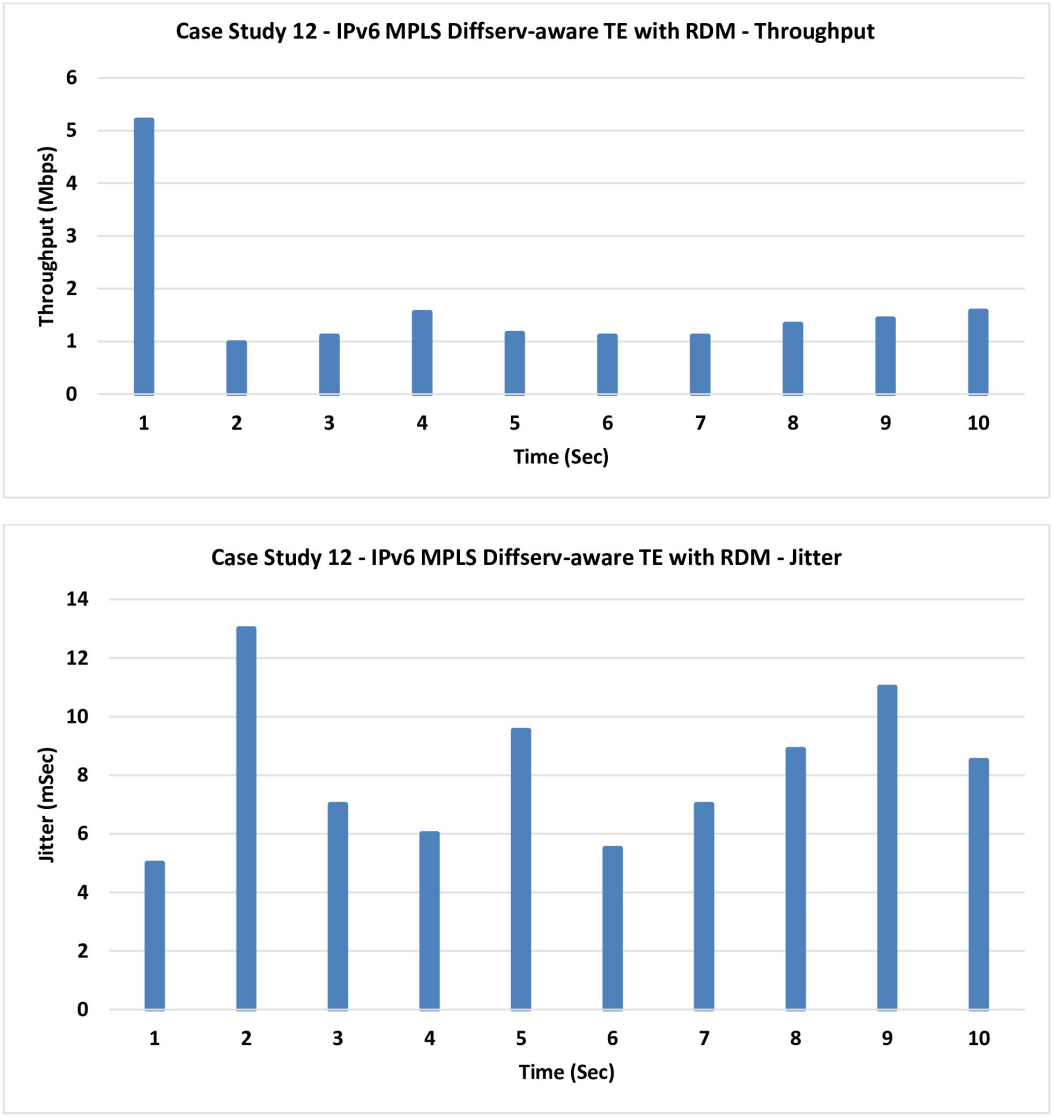
Case Study 12—IPv6 MPLS Diffserv-aware TE with RDM.

The obtained results in Fig 19 are very similar to those of the previous case with a slight improvement as the previously mentioned congestion still exists, and it requires an avoidance technique in the data plane as the next case introduces.

**13. Case Study 13**
  - **IPv6 MPLS Diffserv-aware TE with MAM and QoS LLQ:** In this case, LLQ as a QoS technique is tested with MAM as the bandwidth allocation model.

**Fig 20:**
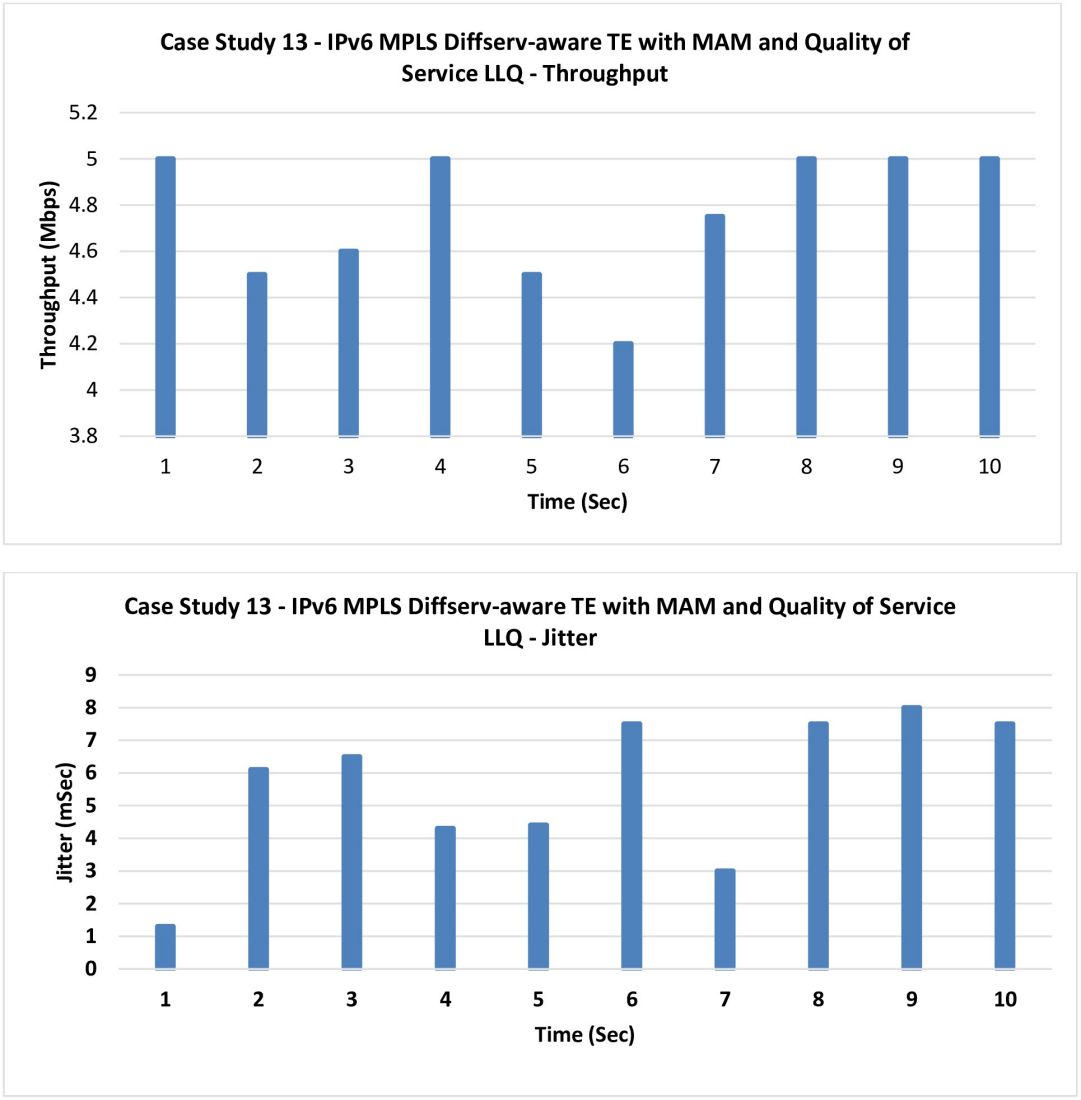
Case Study 13—IPv6 MPLS Diffserv-aware TE with MAM and QoS LLQ.

A significant improvement can be noticed in Fig 20 after introducing LLQ, which prioritizes the traffic based on its type (here the VoIP is prioritized).

**14. Case Study 14**
  - **IPv6 MPLS Diffserv-aware TE with RDM and QoS LLQ:** In this case, LLQ as a QoS technique is tested similar to the previous case, but with RDM as the bandwidth allocation model.

**Fig 21:**
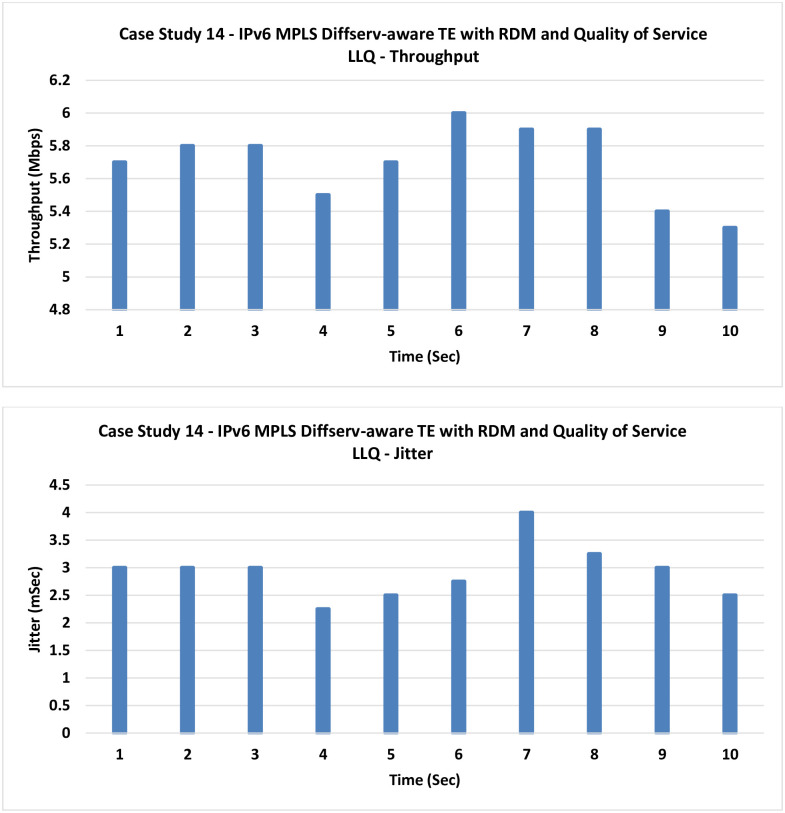
Case Study 14—IPv6 MPLS Diffserv-aware TE with RDM and QoS LLQ.

According to Fig 21, an additional slight improvement can be noticed in the final one of the IPv4 case studies, and this is the proposed model which merges both MPLS-TE Diffserv-aware using the RDM with the LLQ QoQ technique.

## 4. Results and discussion

### 4.1 Insights from prior research findings

The outcomes presented in Dillon Czerny’s study [28] underscore the substantial impact of transitioning from MPLS to MPLS-TE, particularly in terms of mitigating Jitter. Furthermore, the transition to MPLS TE-DS yields additional performance enhancement. A similar trend emerges from the preceding case studies conducted within the framework of this research. However, disparities in virtualization, hardware configurations, and environmental conditions contribute to variations in the outcomes. It is worth noting that the absolute values of the prior research cannot be directly compared to those of the proposed model due to the different hardware setups employed in the case studies, consequently exerting a pronounced influence on the outcomes. Hence, the technique employed in the literature was reassessed using the identical hardware configuration as that with the proposed model, enabling a more accurate comparison of results as indicated by Tables 2, 3 and 4.

**Table 2:** Findings derived from the preceding table.

#	Case Study	Bit Rate (Kbps)	Jitter (mSec)
**1**	MPLS	0.64	235.097
**2**	MPLS TE	0.64	182.772
**3**	MPLS TE-DS	0.72	72.80

**Table 3:** IPv4 different scenario results.

	Case Study	Throughput (Mbps)	Jitter (mSec)
1	IPv4 Traditional Routing	0.7625	15.65
2	IPv4 QoS LLQ.	1.21875	10.15
3	IPv4 MPLS	1.17	10.45
4	IPv4 MPLS Diffserv-aware TE with MAM	1.61175	9.2125
5	IPv4 MPLS Diffserv-aware TE with RDM	1.7	8.575
6	IPv4 MPLS Diffserv-aware TE with MAM and QoS LLQ	4.7875	5.3125
7	IPv4 MPLS Diffserv-aware TE with RDM and QoS LLQ	5.8375	3.037

**Table 4:** IPv6 different scenario results.

#	Case Study	Throughput (Mbps)	Jitter (mSec)
8	IPv6 Traditional Routing	0.69125	15.5
9	IPv6 QoS LLQ.	1.235	9.2
10	IPv6 MPLS	1.065	11.95
11	IPv6 MPLS Diffserv-aware TE with MAM	1.3675	8.975
12	IPv6 MPLS Diffserv-aware TE with RDM	1.65	8.14
13	IPv6 MPLS Diffserv-aware TE with MAM and QoS LLQ	4.755	5.61
14	IPv6 MPLS Diffserv-aware TE with RDM and QoS LLQ	5.7	2.925

### 4.2 Comparison tables (IPv4)

Table 3.

### 4.3 Comparison table IPv6

Table 4.

### 4.4 Throughput (bit rate) comparison for IPv4

**Fig 22:**
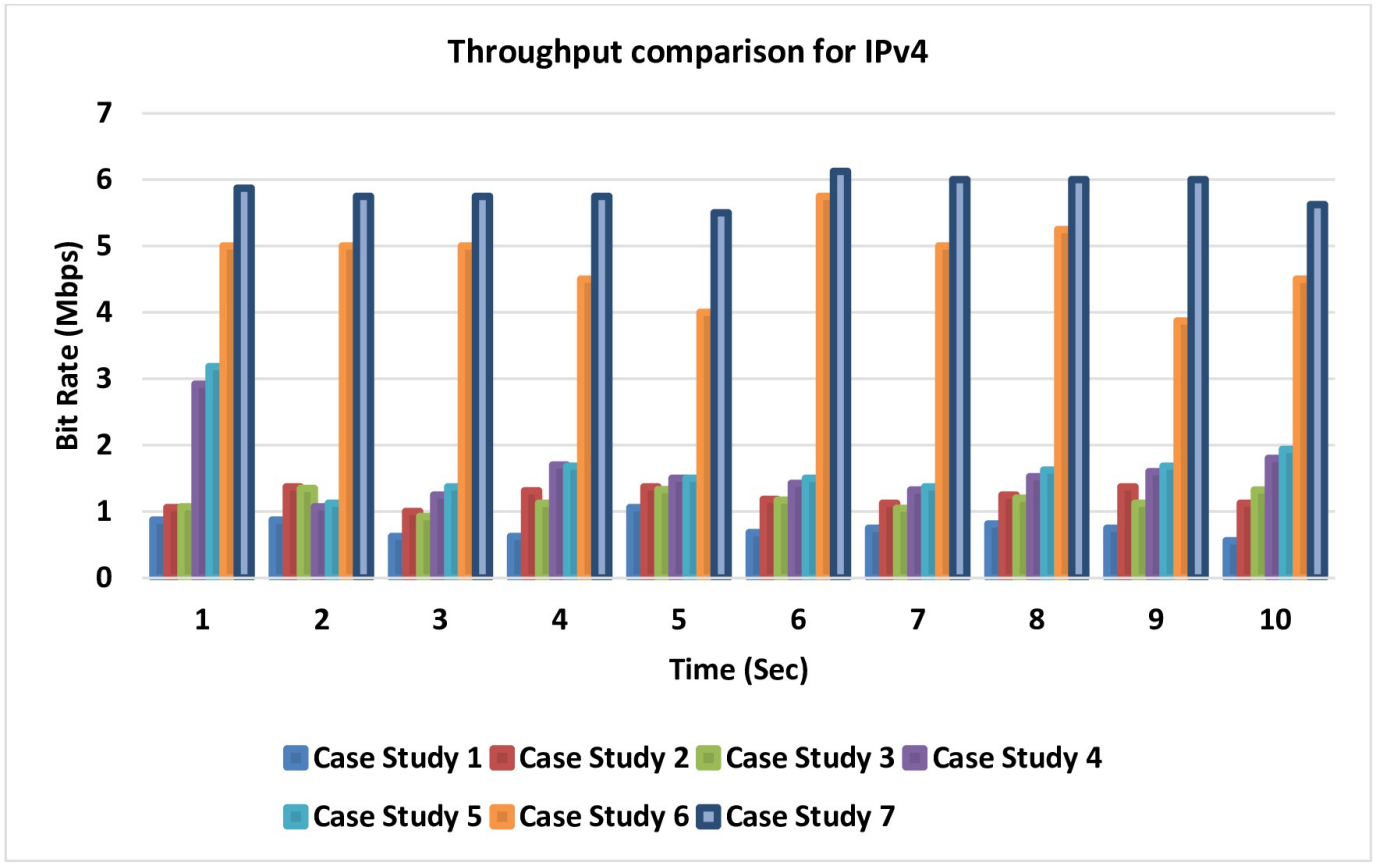
Throughput comparison for IPv4.

Fig 22 presents the throughput comparison for IPv4. It is noticed from this comparison for the seven cases related to IPv4 that there is a difference in the throughput that could be gained in each case study. The largest bit rate which is the best result is noticed in cases 6 and 7 after adding the LLQ to the MPLS-TE.

### 4.5 Jitter comparison for IPv4

**Fig 23:**
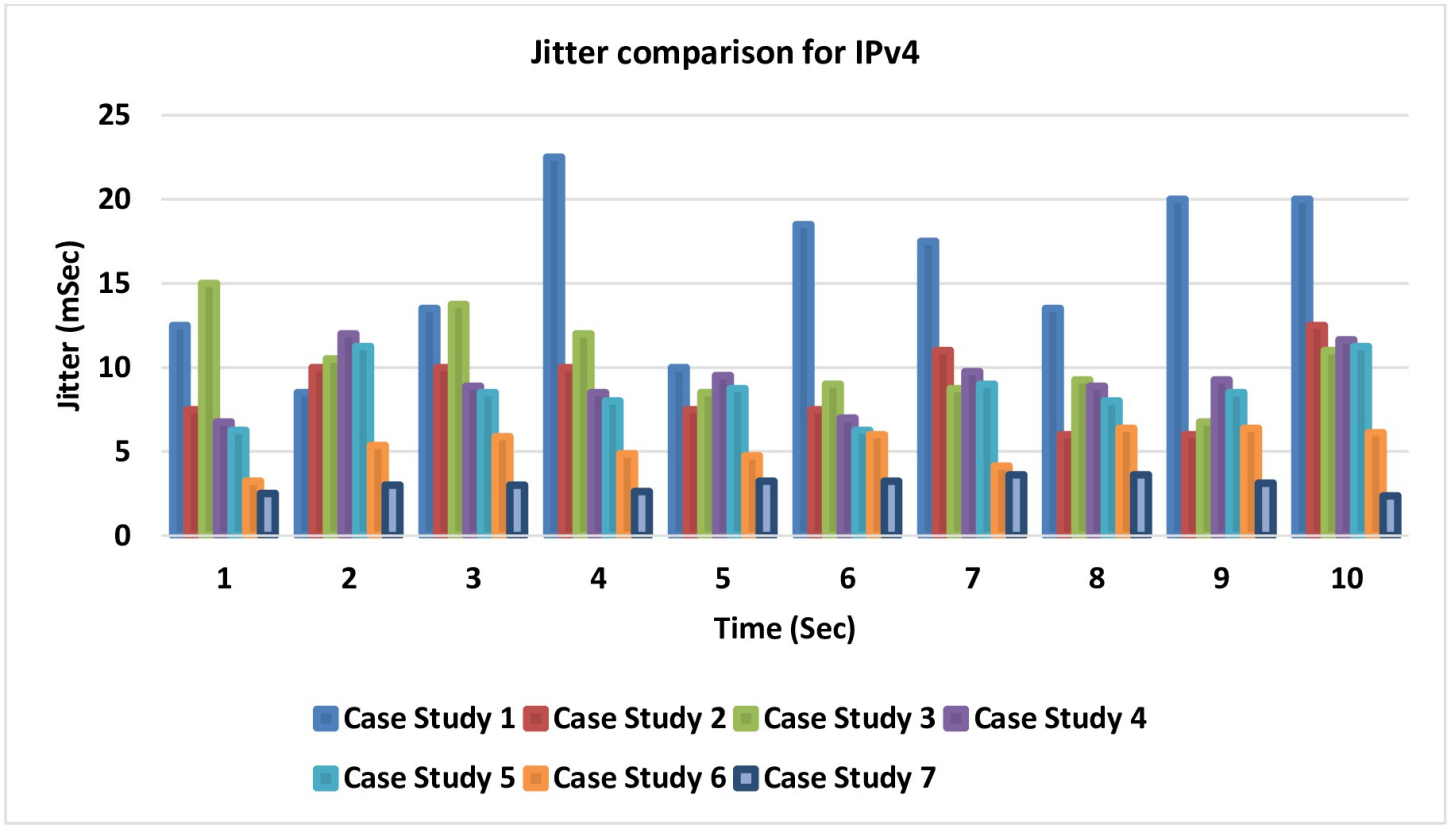
Jitter comparison for IPv4.

Fig 23 introduces a comparison for the seven cases related to IPv4 for the differences in the Jitter that could be gained in each case study. The smallest amount of Jitter, which is the best result, is noticed in cases 6 and 7 after adding the LLQ to the MPLS-TE.

### 4.6 Throughput (bit rate) comparison for IPv6

**Fig 24:**
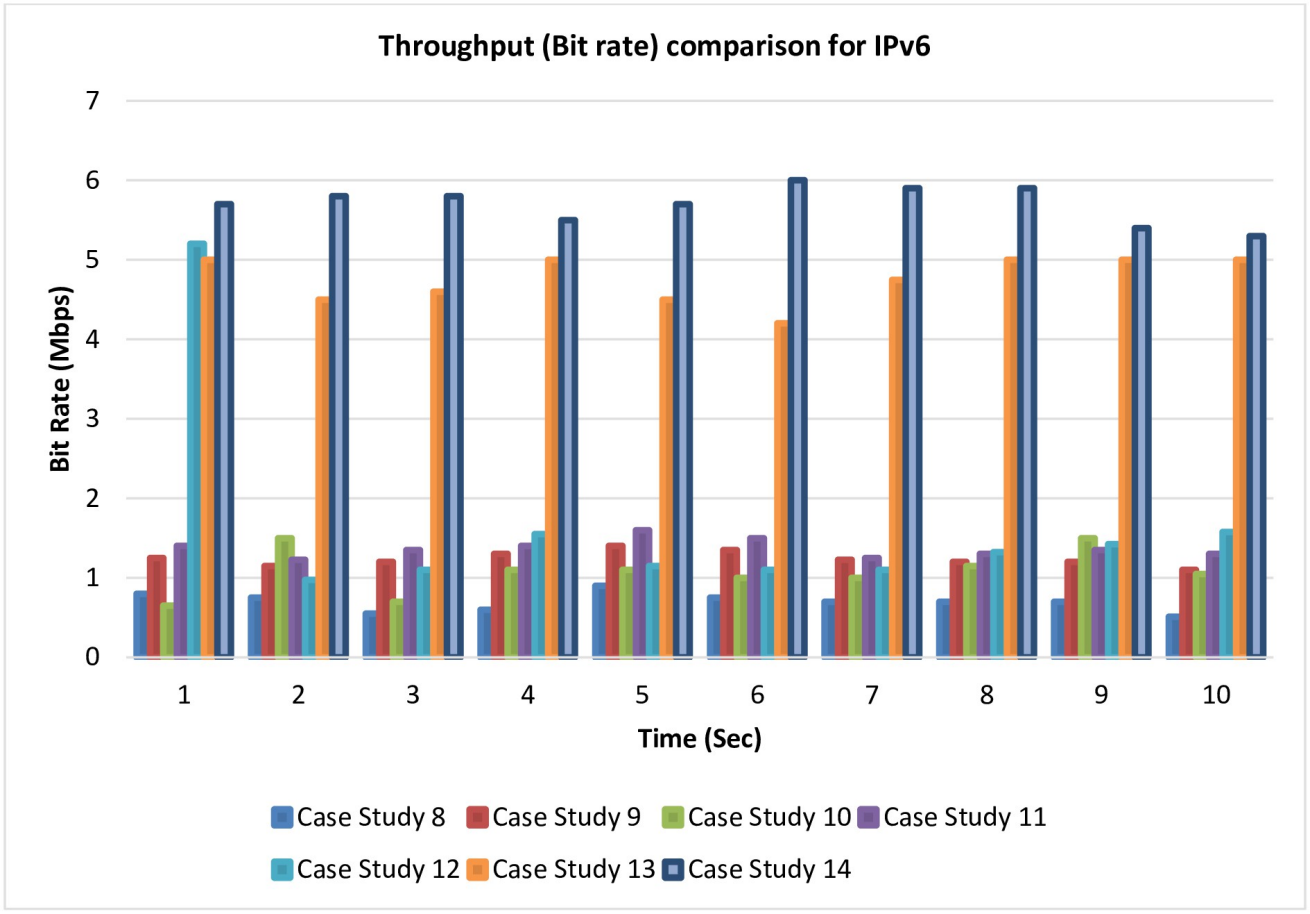
Throughput comparison for IPv6.

Fig 24 outlines the throughput comparison for IPv6. It is noticed from the following comparison for the seven cases related to IPv6 that there is a difference in the throughput that could be gained in each case study. The largest amount of bit rate, which is the best result, is noticed in cases 13 and 14 after adding the LLQ to the MPLS-TE.

### 4.7 Jitter comparison for IPv6

**Fig 25:**
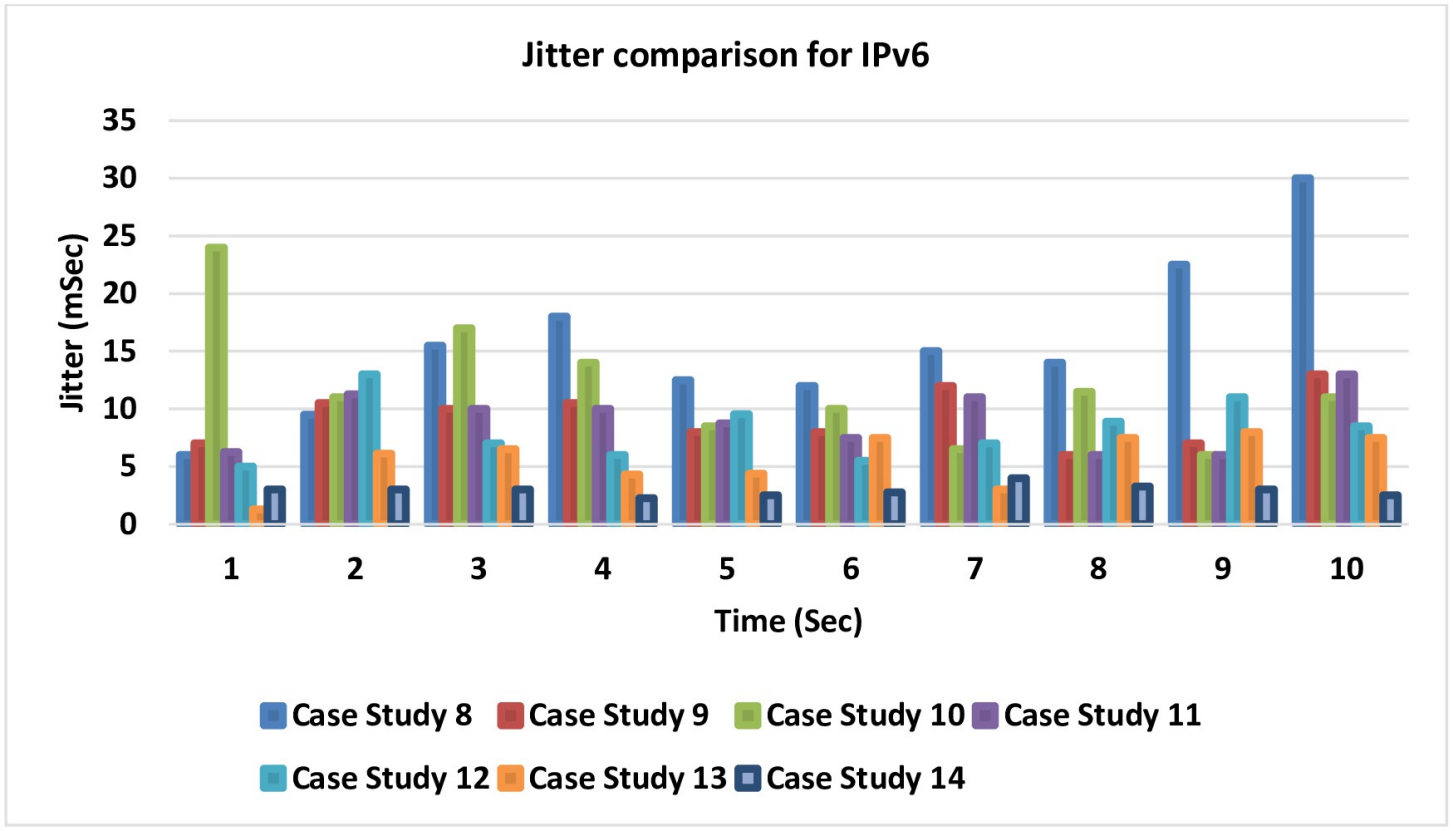
Jitter comparison for IPv6.

Fig 25 introduces a comparison for the seven cases related to IPv6 for the differences in the Jitter that could be gained in each case study. The smaller amount of Jitter, which is the best result, is noticed in cases 13 and 14 after adding the LLQ to the MPLS-TE.

### 4.8 Throughput (bit rate) for IPv4 versus IPv6

**Fig 26:**
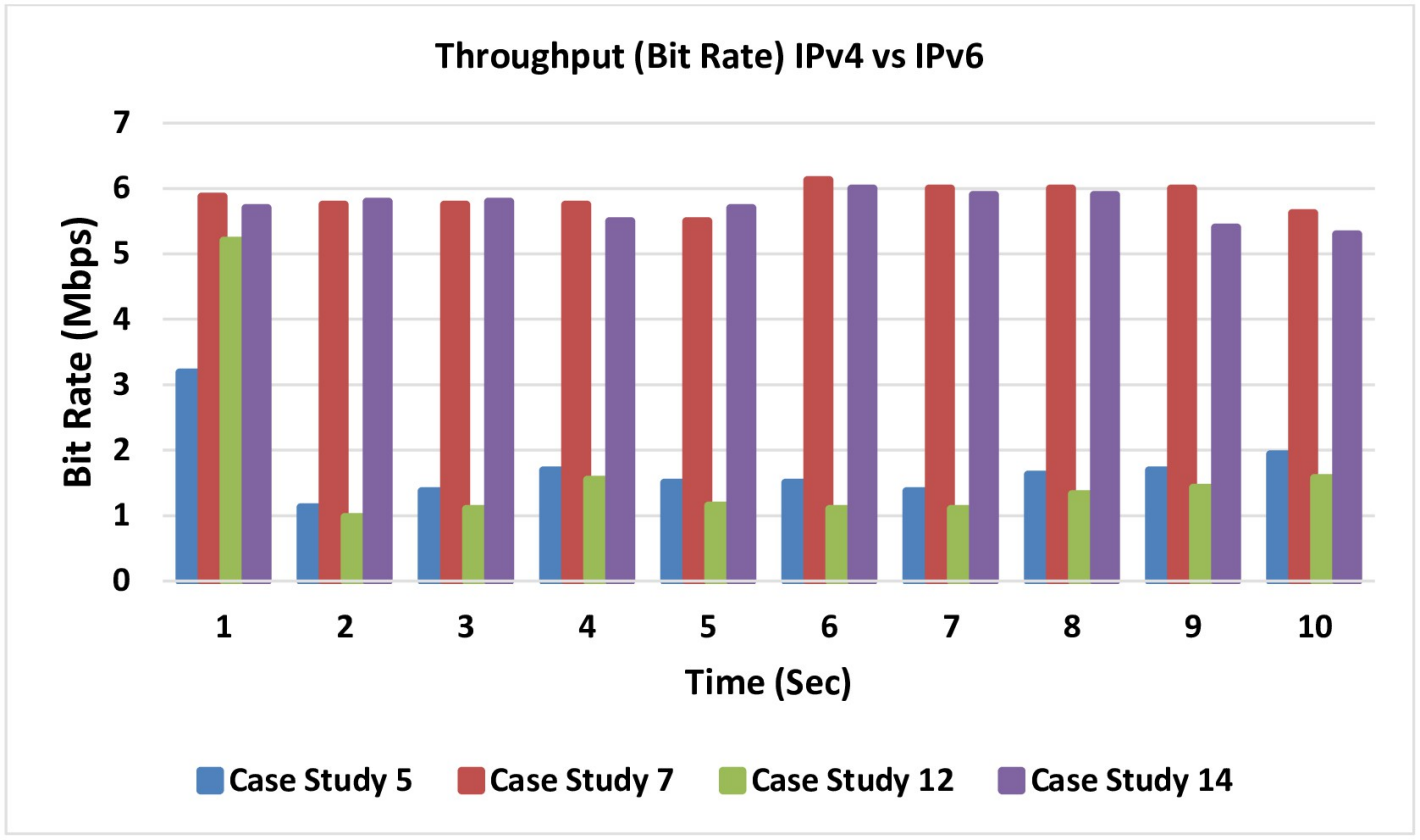
Throughput comparison between IPv4 and IPv6.

Fig 26 presents a throughput comparison between IPv4 and IPv6. This serves as a juxtaposition between IPv4 and IPv6, revealing marginally superior results for IPv4. This discrepancy can be attributed to the variance in header sizes between the two protocols; the larger IPv6 header exerts a minor influence on network resource utilization.

Case studies 5 and 12 showcase the methodology aligned with the literature, involving MPLS-TE with RDM, omitting LLQ. Conversely, case studies 7 and 14 exemplify the proposed model, namely fusion of MPLS-TE with DiffServ awareness and QoS LLQ.

A conspicuous enhancement becomes evident, when comparing the proposed model to the traditional approach. The proposed model yields a noteworthy increase in the available bandwidth, enabling a greater number of concurrent VoIP calls with improved quality.

### 4.9 Jitter for IPv4 versus IPv6

**Fig 27:**
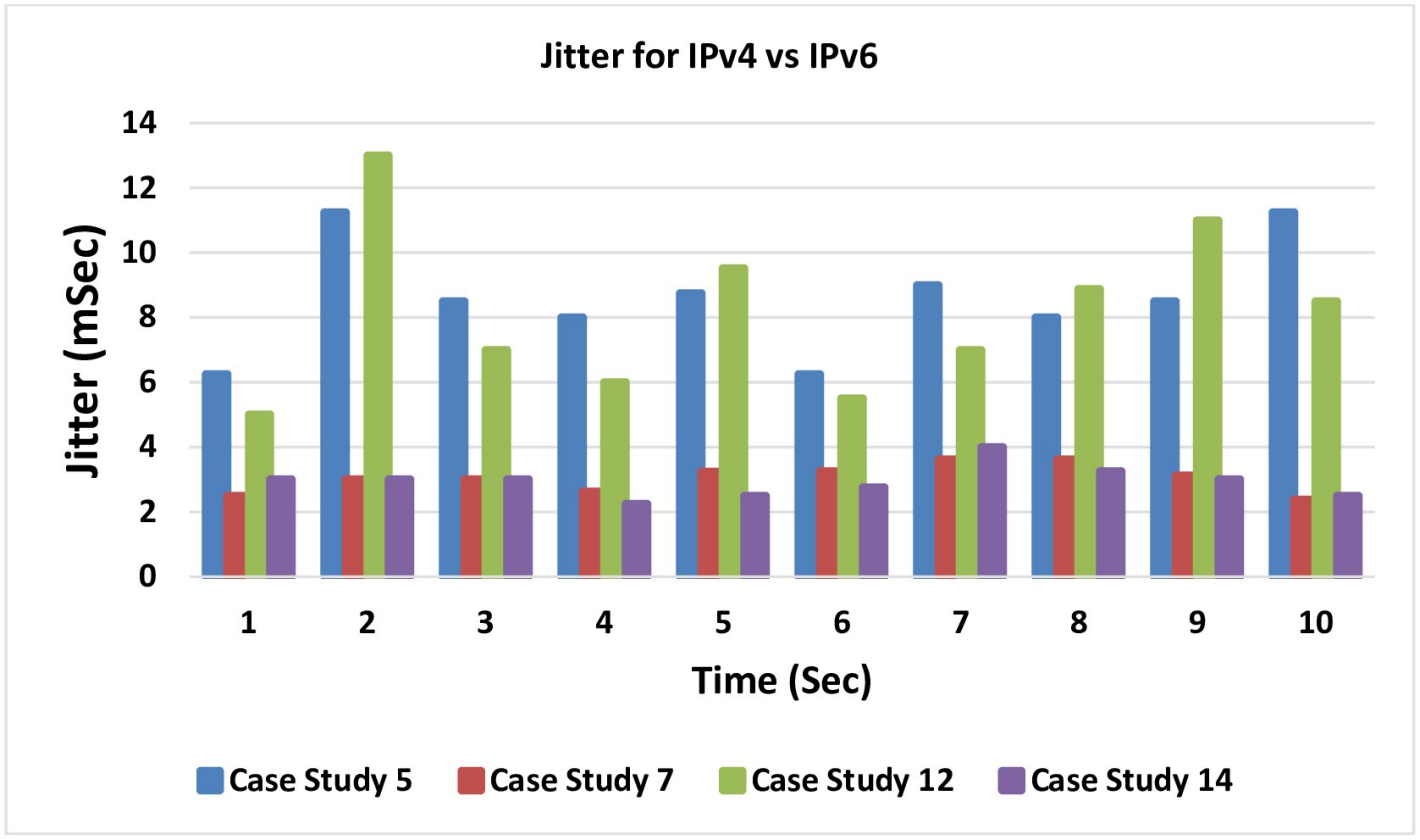
Jitter comparison between IPv4 and IPv6.

Jitter comparison between IPv4 and IPv6 is presented in Fig 27. This analysis entails a juxtaposition of IPv4 and IPv6 outcomes, revealing a slight favorability towards IPv4. This tendency finds explanation in the different header sizes inherent to the two protocols; the larger IPv6 header mildly influences network resource utilization.

Case studies 5 and 12 exemplify the approach adopted in the literature, involving MPLS-TE with RDM but excluding LLQ. In contrast, case studies 7 and 14 embody the proposed model, an amalgamation of MPLS-TE with DiffServ awareness and QoS LLQ.

A notable enhancement surfaces when comparing the proposed model to the traditional approach. The proposed model substantially reduces Jitter in traffic, culminating in a network capable of delivering significantly improved VoIP services with enhanced quality.

## 5. Conclusion

This study has thoroughly explored the escalating demand for high-bandwidth Internet connections, shedding light on the challenges faced by ISPs in effectively managing bandwidth and network traffic. To address these challenges, we introduced a synergistic approach by integrating MPLS-TE with Diffserv-QoS technologies that extend beyond conventional networks and find profound resonance in the domain of smart agriculture. The increasing reliance on technology in agriculture, driven by real-time data demands, remote monitoring, and automated processes, underscores the necessity for robust and reliable high-bandwidth connections. Our proposed synergy optimizes bandwidth utilization through MPLS-TE and deploys traffic control mechanisms with Diffserv-QoS, providing ISPs with the tools to create a resilient network foundation for smart agriculture applications. Notably, the integration of MPLS-TE with Diffserv-QoS has yielded substantial improvements, enhancing throughput and significantly reducing Jitter. Utilizing the IPv4 header, we achieved impressive outcomes, reaching a throughput of 5.83 Mbps and reducing Jitter to a remarkable value of 3 msec. This study not only addresses immediate network challenges but also lays the groundwork for a more connected and advanced digital landscape, particularly in the realm of smart agriculture.
