# Correction: Optimizing bandwidth utilization and traffic control in ISP networks for enhanced smart agriculture

**DOI:** 10.1371/journal.pone.0343356

**Published:** 2026-02-23

**Authors:** The PLOS One Editors

The Data Availability statement for this article [1] is incorrect. The correct statement is: The original underlying data to support all results in the article and Supporting Information files are available at Figshare (https://figshare.com/s/48c0e2a4b319b3116be4).

The authors also stated that the AI tool Grammarly was used for grammar checks during the preparation of the manuscript.
